# Magnetic and electric polar regions in the magnetoelectric composite microstructure

**DOI:** 10.1038/s41598-025-20911-z

**Published:** 2025-10-22

**Authors:** K. P. Jayachandran, Deepa Rajendran Lekshmi, J. M. Guedes, K. P. Surendran, H. C. Rodrigues

**Affiliations:** 1 IDMEC, Instituto Superior Técnico, Universidade de Lisboa, Av. Rovisco Pais, 1049-001 Lisbon, Portugal; 2 School of Pure and Applied Physics, Mahatma Gandhi University, Kottayam, 686560 India; 3 Materials Science and Technology Division, CSIR- National Institute for Interdisciplinary Science and Technology Division (CSIR-NIIST), Thiruvananthapuram, 695019 India; 4 Academy of Scientific and Innovative Research (AcSIR), CSIR, Ghaziabad, 201002 India; 5 Present Address: Department of Physics,, Sree Sankara College, Nagaroor, Kilimanoor, Kerala, 695601 India

**Keywords:** Computational methods, Information storage, Magnetic properties and materials, Electronic properties and materials, Ferroelectrics and multiferroics, Computational science

## Abstract

Magnetoelectric (ME) composites containing lead-free oxides with high dielectric constant and switchable electric polarization are required for use in devices in integrated microelectronics and memory storage. Introduction of random local heterogeneity originally meant to enhance ferroelectric polarization is employed in ME composite to generate ME response through simulation and experiment. Using a combination of continuum simulations, atomic force microscopy, and screen printing of ME composite we demonstrate robust magnetoelectric coupling in environmentally benign lead-free rare earth substituted SrTiO$_3$–CoFe$_2$O$_4$ system. Distinct from other reports, the locations of polar magnetic regions (PMRs) and electric regions -that are critical for information storage- in the microstructure are identified in this system. Antiparallel local magnetic field vectors dots the microstructure of the composite. Detection of local stress/strain formations in accordance with magnetostriction is a prime validation of the robustness of computational model. Besides, the computed averages of polarization and magnetization along the applied field direction are found to be in good agreement with the measurements. Histograms of local strains were mapped in order to go incisively into the composite microstructure. Substantial build-up of electrical potential (a measure of the abundance of piezoelectric charges) induced by the external magnetic field is one of the key features observed.

## Introduction

Magnetoelectric (ME) effect, proposed by Landau and Lifshitz in the late 1950s, manifests as a tractable linear relation between magnetic and electric fields in matter. Magnetoelectric multiferroic crystals placed in a constant electric (or magnetic) field, could exhibit magnetic (or electric) moment proportional to the field^1^. This phenomenon, known as magnetoelectric (ME) effect, is responsible for ME coupling when a ferroelectric material is layered with a ferro- or ferrimagnet in a classic horizontal composite architecture^2^. Coupling could usher in a scenario that permit data to be electrically written and read magnetically by exploiting the features of ferroelectric random access memory (FeRAM) and magnetic data storage unlike the independent encoding of information by magnetization and polarization^3^. This can avoid problems associated with reading from FeRAM and prohibitive requirement of large local magnetic fields to encode information. Electric fields, rather than magnetic fields or current, are required to control magnetic moment directions in order to mitigate heating and making it more energy-efficient. Moreover, these materials offer logic states where the data can be stored in both, the electric and magnetic states^3,4^. ME composites containing lead-free perovskite-like oxides with high dielectric constant and switchable electric polarization are required for such memory storage applications^5^. The most obvious strategy to engineer such a system with reasonable coupling and transition temperatures (both magnetic and ferroelectric) involves combining both magnetic and ferroelectric (FE) materials into a ME composite^6^. ME multiferroic fluids composed of FE-ferromagnetic composite nanoparticles too have shown to display elegant ME coupling^7–9^.

**Figure 1:**
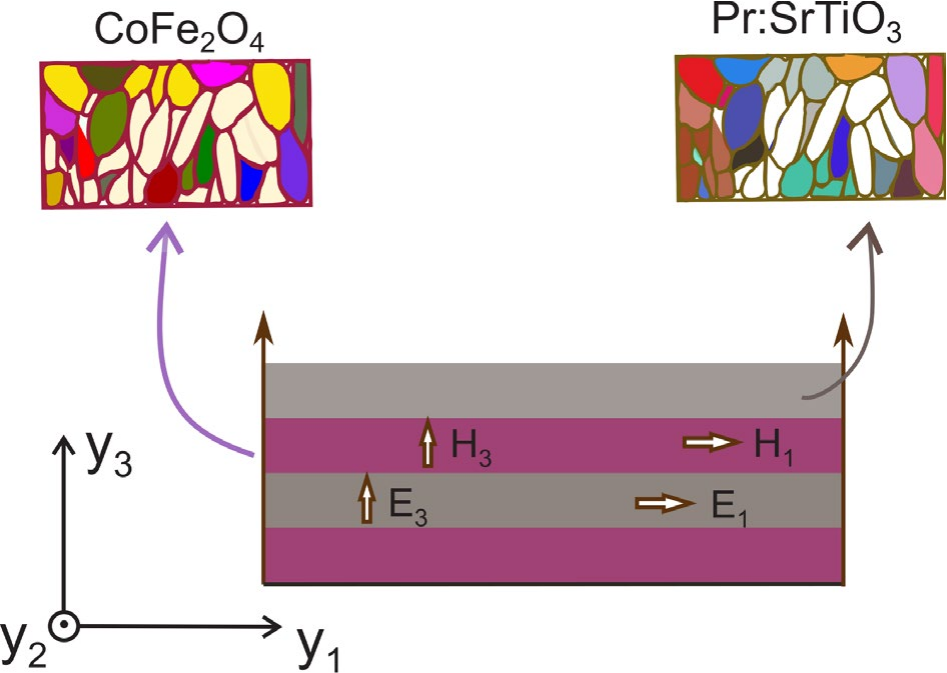
Schematic picture of an ideal multilayer setting for simulation of ME composite constituted by polycrystalline Pr doped STO (i.e., Pr:STO) and CFO. $\mathbf {E_i}$ and $\mathbf {H_i}$ are the components of external electric and magnetic fields. (Different colours of granular substructure of constituent phases represent random polarization orientation (magnetic polarization in CFO and electric polarization in Pr:STO) of the grains.).

Strain-induced magnetoelectric coupling in ferroelectric–ferromagnetic (FE–FM) composites can be achieved by variety of methods^10^. Relevant among them is the stacking of FM and FE layers into composite magnetoelectric laminate (see Fig. 1) where the strain is transmitted between phases relying on the bonding between the layers^11^. Since the voltage control of magnetism in magnetic metals are overshadowed by the short screening lengths, researchers explore the efficacy of magnetic/ferroelectric oxide bilayers^12,13^. The hysteretic electric field-polarization profile of the ferroelectric material can help hold on the magnetization in contrast to the FM/dielectric interface where the magnetization disappears once the electric field is switched off. Spinel magnetic oxides such as CoFe_2_O_4_ (CFO) possesses magnetic transition temperature ($T_N$) much higher than room temperature and is having good coherence with FE oxide SrTiO_3_ (STO) in the crystal structure that pave the way for preparation of high-quality magnetoelectric films. The FE phase is poled near the ferroelectric Curie temperature ($T_C$) in a strong electric field to derive the composite piezoelectric while the magnetic poling of the ferromagnetic (FM) phase is accomplished in a similar way, by annealing the composite in a magnetic field near the Néel temperature ($T_N$).

Significant changes in the magnetic properties of the ferromagnet could be attained by means of strain transfer across interface (magneto-elastic effect) in an FM/nonmagnetic bilayer setting. Our approach is to replace the nonmagnetic layer in such settings with FE, and thereby the strain transfer can be reinforced through the induction by an electric field, thanks to the inverse piezoelectric effect prevails in the FE material. This additional strain transfer allows one to manipulate the magnetisation by an electric field^14^. High dielectric constant of STO, its nonlinear electric-field dependence, softening of elasticity due to temperature and its perovskite structure make it a unique candidate for multilayer ME composite^15^.

Donor doping- replacing the A and/or B-site cations with a small amount of higher valency cations- could sow disorder due to random defect distribution that can eventually enhance piezoelectric properties^16,17^. Landau’s phenomenological theory demonstrate that magnetization is coupled with electric field via inverse magnetoelectric coupling constant $\alpha$ which paves the way for modulation of magnetization by electric field^14^. The ME coupling is arguably through strain transfer from FE layer to ferromagnetic layer which will induce (or enhance) magnetic properties in this system. Because of the inverse piezoelectric effect, the electric field applied to the FE material strains it and that gets migrated to the FM phase. This will eventually produce a magnetization due to piezomagnetism. Thus materials engineered to possess high piezoelectricity would suffice for better ME coupling in composite setting^18^. Recent compilation of the experimental (and some theoretical) works on voltage control of magnetism focussing on thin film/thick film heterostructures^14,19^ underscore the need for magnetic/electric domain location mapping of the ME composites.

Randomness, introduced by local microstructural heterogeneity could potentially enhance piezoelectricity in relaxor ferroelectric ceramics^17,20,21^. As previously shown, introduction of heterogeneity locally can improve the piezoelectric properties of FE materials and the ensuing coupling effect of the ME composite of which the former is a component^17,22^. Here, we have incorporated inhomogeneity in the simulation of incipient ferroelectric SrTiO$_3$ (STO) phase (of the layered SrTiO$_3$–CoFe$_2$O$_4$ composite) through randomness in the grain orientations and accomplish the same experimentally by cation (Pr) doping in STO. The local magnetization regions, build up as a consequence to the electric field, where the fingerprints of magnetic storage locations are situated are mapped. These regions called *polar magnetic regions* (PMRs), which would be defined soon, manifest itself through the local magnetic quantities *viz.,* magnetic-fields, -fluxes or -potential distributions. Vector $\textbf{H}$, the magnitude of magnetic field strength is independent of the permeability of the medium while magnetic flux density $\textbf{B}$ is dependent on the permeability of the medium. In this particular viewpoint, vectors $\textbf{H}$ and $\textbf{B}$ are analogous to vectors $\textbf{E}$ and $\textbf{D}$ respectively. Hence $\textbf{B}$ and $\textbf{H}$ are measures of magnetization vector occurring in the material^23^. PMRs are thus small regions in the microstructure spanning one or more crystallographic grains where the local magnetization is uniform in a polycrystalline ME composite. Since the dimension of a typical PMR can exceed that of crystallographic grain, these regions are distinct from the customary subgranular ferromagnetic domains^24^. In the present work, the non-magnetic incipient ferroelectric STO is combined with the non-FE yet magnetostrictive CoFe$_2$O$_4$ (CFO) to exhibit this “product property”- ME effect- which neither of the phases possess individually^25^. Moreover, the homogenization procedure employed in this system allows us to explore a wealth of microscopic magnetic and electric field locations that were previously inaccessible. Yet, the parallels drawn between simulation and experiment in this study would not be complete until refined electronic structure calculations are performed in this system as a future task.

## Simulation by homogenization

In order to establish the concept of enhancement of piezoelectricity and thereby ME coupling by the introduction of local inhomogeneity and to mapping the electric and magnetic regions, we first performed a simulation study using homogenization method through a two-scale asymptotic analysis of the microscopic electric, magnetic and mechanical fields^26,27^. The unit cells are so chosen that they can encompass the heterogeneity of the composite statistically. The magnetoelectric effect is described by a term in the thermodynamic potential $\Phi _{{\textbf {me}}}$ that is linear both in the magnetic (${\textbf {H}}$) and electric (${\textbf {E}}$) field, such that

1 $$\begin{aligned} \Phi _{{\textbf {me}}}=-\alpha _{ik}E_iH_k \end{aligned}$$

where the magnetoelectric coefficient $\alpha _{ik}$ is an unsymmetrical tensor^28^. When ${\textbf {H}}=0$, the electric field generates a magnetization $M_{k}=\alpha _{ik}E_{i}$ and when ${\textbf {E}}=0$, the magnetic field generates an electrical polarization $P_{i}=\alpha _{ik}H_{k}$. Further details are seen in the Methods section. Local and average (global) electrical, magnetic, and mechanical constitutive behaviour are computed. For a linear magneto-electro-elastic solid, constitutive equations are governed by the electrical, mechanical and magnetic fields. The model quantifies the local electrical and magnetic potential, displacements, electrical and magnetic fields, stress and strain fields, magnetization (through magnetic flux density) and polarization (through electric displacement) and von-Mises stress, besides the effective magneto-electro-mechanical properties.

We simulate a polycrystalline ME composite with orientational discontinuity of grains constituting the FE phase (STO) in order to introduce disorder at the microstructural level. Experimentally, it can be achieved by donor doping-i.e., exchange of A-site (sometimes B-site as well) cations with a small amount of cations having higher valence. Here the doped STO polycrystal acquires ferroelectric properties and consequently polarization is built up in doped STO grains. This polarization is discontinuous across the granular FE structure and we introduced a small degree of disorder in the orientation distribution. This could simulate the donor doped STO phase in the ME composite. The resulting energy landscape become flatter so that the FE and FM phases can interlink seamlessly. The equivalent properties of the polycrystalline ME composite and the influence of macroscopic average fields acting on it can be described using asymptotic analysis of multiferroic ME material. This treatment also leads to the product property of the homogenized ME coupling $\widetilde{\alpha }_{ij}$^29^. The homogenized magnetoelectric properties thus obtained (Table  1) are comparable to other ME composites of similar architecture in literature^30^. Since the individual phases of the composite being devoid of any multiferroic property, they lack the property of ME coupling as such. Nevertheless, the ME composite resulted from juxtaposing the incipient ferroelectric STO and magnetostrictive CFO show this product property of coupling as is shown Table 1. It is possible to obtain the effective ME voltage coefficient (or the ME coupling coefficient (MECC)) $\alpha _{E}=\delta E/\delta H$ through the knowledge of $\widetilde{\alpha }$ in such a way that $\widetilde{\alpha } =\delta P/\delta H=\alpha _{E}\kappa _0\widetilde{\kappa }$ of the composite^31^. Here, E, P and $\kappa _0$ are the electric filed, polarization and permittivity of free space respectively. $\widetilde{\kappa }$ is the effective relative permittivity of the composite. Since we do not have experimental characterization of full ferroelectric data, we have used computational data for STO from Erba et al.,^32^, where they used *ab initio* theoretical simulation. However, the deviation of computational value of ME voltage coupling $\alpha _{E}$ is expected mainly due to the paucity of Pr:STO to be used for simulation. Notwithstanding this, the effective values given in Table 1 for the ME composite would provide a guide to experimental characterization of STO–CFO composite.

We computed the effective $\alpha _{E}$ using the approach developed in Ref.^31^ and found a value of $\alpha _{E} =52.7$ mV/(cmOe) which is comparable with the present 96 mV/(cmOe). The strong ME coupling is expected in a layered structure primarily due to low leakage currents and ease of poling to align the electric dipoles and thereby strengthen the piezoelectric effect of the ferroelectric phase of the composite^33^. We have computed the homogenized averages of the properties of the single-crystalline ME composite and found that the ME coupling values values are significantly lower compared to the polycrystal values reported in Table  1. (last section of the Supplementary material for the single crystal results). For instance, $\alpha _{E}<1$ mV/(cmOe) and $\alpha _{E11}\approx 6$ mV/(cmOe) in single crystal STO–CFO composite. This observation corroborates the notion of the significance of local heterogeneity in enhancing the coupling in ME composites.

**Table 1:** Values of the homogenized piezoelectric stress coefficients $\widetilde{e}_{i\mu }$ (in C/m$^2$), piezomagnetic coefficients $\widetilde{e}^{~M}_{i\mu }$ (in N/Am) and dielectric permittivity $\widetilde{\kappa }^{\epsilon H}_{ij}$ (in $\kappa _0$), ME coupling $\widetilde{\alpha }_{11}$ (in 10$^{-8}$ Ns/VC ), $\widetilde{\alpha }_{33}$ (in 10$^{-10}$ Ns/VC ), and ME voltage coefficients (absolute value) $\widetilde{\alpha }_{E 11}$ (in mV/cmOe), $\alpha _{E}$ (in mV/cmOe) of ME composite STO–CFO. ($\kappa _0$ is the permittivity of free space).

$\widetilde{e}_{31}$	$\widetilde{e}_{33}$	$\widetilde{e}_{24}$	$\widetilde{e}^{~M}_{31}$	$\widetilde{e}^{~M}_{33}$	$\widetilde{e}^{~M}_{24}$	$\widetilde{\kappa }^{\epsilon H}_{11}$	$\widetilde{\kappa }^{\epsilon H}_{33}$	$\widetilde{\alpha }_{11}$	$\widetilde{\alpha }_{33}$	$\widetilde{\alpha }_{E11}$	$\alpha _{E}$
0.02	0.18	0.80	23.8	36.3	397.9	127.2	20.3	-0.48	-0.22	3418.6	96

**Figure 2:**
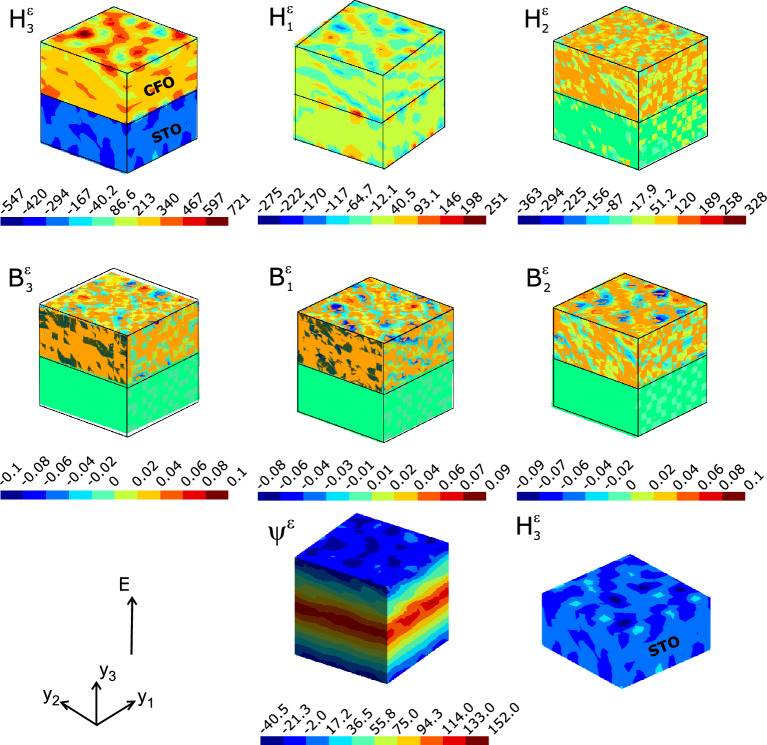
Fingerprints of magnetic regions in the composite microstructure of polycrystalline SFO–CFO upon the application of electrical field. Local magnetic field ($H_j^\varepsilon$ in A/m), the out-of-plane component $H_3^\varepsilon$ and the inplane components $H_1^\varepsilon$ and $H_2^\varepsilon$ are shown. The magnetic flux densities $B_j^\varepsilon$ and the magnetic potential $\psi ^\varepsilon$ are shown.

## Results

### Application of electric field

Here the distribution of microscopic fields computed at every nodal points of the finite element mesh (FEM) constructing the microstructure of STO–CFO composite upon applying an external electric field $\mathbb {E}$. The volume fraction of the FE (STO) component in the ME composite is kept at $v_f=50\%$ (or equivalently $v_f$ of CFO is 50%). The lower layers in the subplots shown in Fig. 2 refers to the STO layer and the top layer is CFO. The spread of local fields provide an indication of the response of the material microstructure to the applied electric field. The advent of magnetic regions is an important finding since the use of a data write scheme based on the application of a voltage is proposed as an accepted method that drastically reduce the writing energy of MRAMs (Magnetic Random Access Memories)^12^. Here the sites of the magnetic vectors $\textbf{H}$ and $\textbf{B}$ are arguably *polar magnetic regions (PMRs)*. Here we found magnetic field distributed strongly but staggered along the out-of-plane ($H_3^\varepsilon$) direction of the composite lamina compared to the inplane fields (top panel of the Fig. 2). In the CFO layer, we could identify regions with negative magnetic polarization (the cyan coloured patches in Fig. 2). Moreover, a reversal of direction of $H_3^\varepsilon$ is seen across the STO layer as is seen in a detailed view of the field distribution along STO layer at the bottom panel of Fig. 2.

The magnetic flux $B_i^\varepsilon$ shown in Fig. 2 exhibits the normal component of the magnetic field thus developed consequent to the electric field on ME composite system. Since the magnetoelectric coupling resulting from $\mathbb {E}$ in ME composites is facilitated through stress/strain transfer due to piezoelectric effect in the FE phase and the consequent straining of the FM layer. This would result in the generation of a magnetic potential and hence the magnetization of the composite. The magnetic scalar potential $\psi ^\varepsilon$, exhibits the advent of magnetism consequent to the application of electric field, obviously due to the magnetoelectric coupling appearing in the composite. It is a vital parameter that the $\psi ^\varepsilon$ and its distribution can provide information regarding the magnetic field (see the Eq. S7 in Supplementary material for its relationship ) and its distribution across the composite. The magnetic potential $\psi ^\varepsilon$ shows a concentration near and across the interface. This is arguably due to the lattice mismatch (though it is not explicitly expressed in the model) between CFO and STO that can eventually spur the ambient magnetic ordering as is evident in interface-driven ME coupling in other perovskite composites^34^.

We have analysed the local stress and strain profile of the composite microstructure in order to ascertain the mediatory role played by it in the coupling mechanism (Supplementary Fig. S7). The microscopic stress profile $\sigma _{ij}^{\varepsilon }$ shown in Fig. S6 exhibits the distribution of this stress and its values provide a measure of the strength of the coupling. It is seen that the components $\sigma _{11}^{\varepsilon }$ and $\sigma _{22}^{\varepsilon }$ characterising the distribution along the *xy*–plane of the composite show stress contraction along the magnetic layer (-ve values). The shear components ($\sigma _{12}^{\varepsilon }$ and $\sigma _{13}^{\varepsilon }$) show nearly uniform distribution. Similar is the spread of longitudinal stress component $\sigma _{33}^{\varepsilon }$ along the *z*–axis of the composite. Expansive strain along the *z*–axis across the FE layer ($\epsilon _{33}^{\varepsilon }$) results in an overall negative magnetic field ($\textrm{H}_3^{\varepsilon }$) in that direction. This in accordance with the equation Eq. S3 of the Supplementary material. The observation of nearly an order of magnitude rise in displacement across the composite interface (see the Fig. S6 in Supplementary material ) complements the potential concentration near the interface.

**Figure 3:**
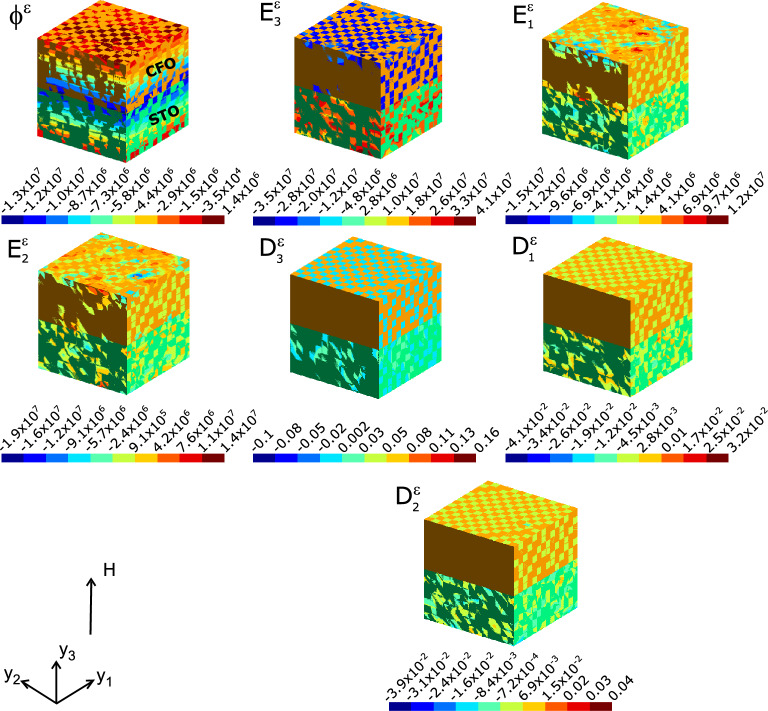
Map of local electric potential $\varphi ^\varepsilon ~(V)$, electric field $\textrm{E}_j^{\varepsilon }~(V/m)$, and electric displacement $\textrm{D}_j^{\varepsilon } (C/m^2)$, computed at the nodal points of the FEM, upon applying a biasing magnetic field $\mathbb {H}$ on a magnetoelectric composite of STO–CFO.

### Application of magnetic field

In contrast to the electrical bias, here we discuss the distribution of local fields in response to an external magnetic field as it simulates magnetic field tuning of the ME composite. Substantial electrical potential ($\varphi ^\varepsilon$) is built up in the composite due to external magnetic field as is seen in Fig. 3 besides the spread of magnetic potential $\psi ^\varepsilon ~$ (given in Supplementary Fig. S8). $\varphi ^\varepsilon$ build-up measures the electrical charges piezoelectrically induced by STO and spread throughout the composite. Apart from this, it is indication of the corresponding fields developed in the composite, since the space variables of the potential yields the fields. The induced electric fields are strong and contrasting in magnitudes. The components $\textrm{E}_1^{\varepsilon }$ and $\textrm{E}_2^{\varepsilon }$ (both having values of the order of $\approx 10^6 V$) are spread nearly uniformly throughout the composite. However, the longitudinal field $\textrm{E}_3^{\varepsilon }$ (which incidentally is the one along the direction of the applied $\mathbb {H}$) permeates into the composite and contrastingly and non-uniformly spread across the different phases of the composite (middle panel of Fig. 3). Moreover, $\textrm{E}_3^{\varepsilon }$ builds up in opposite directions in the composite constituents. The electric displacement vector $\textbf{D}$ is a linear combination of polarization $\textbf{P}$ and electric field $\textbf{E}$ by $\textbf{D} = \kappa _0\textbf{E} + \textbf{P}$, where $\kappa _0$ is the permittivity of free space. The electrical displacement profile thus displays a measure of polarization in different orientations of the composite and is very prominent along the out-of-plane direction of the composite plane where the magnetic field is applied.

**Figure 4:**
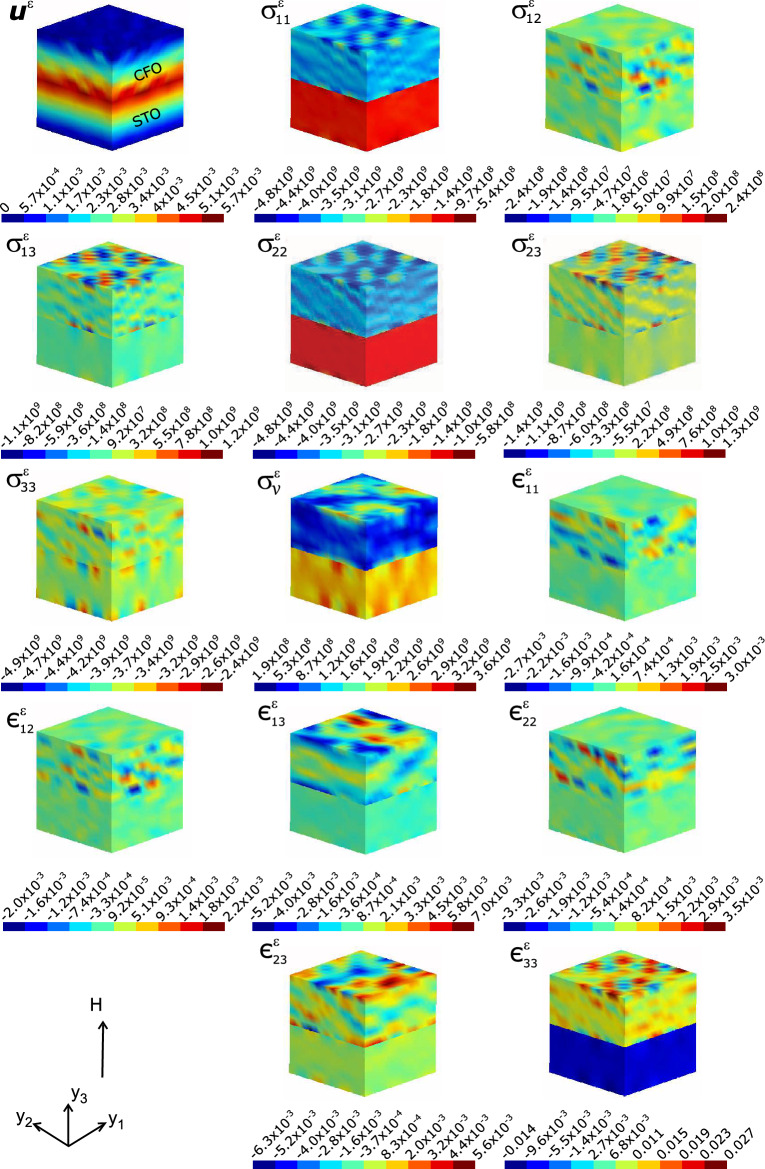
Map of local fields (computed at the nodal points of the FEM) of magnetoelectric composite STO–CFO, *viz.*, displacement $\textbf{u}^{\varepsilon }$, the stress $\sigma _{ij}^{\varepsilon }~(N/m^2)$, the equivalent, von Mises stress $\sigma _v^\varepsilon$ and strain $\epsilon _{ij}^{\varepsilon }$ upon applying a global magnetic field $\mathbb {H}$ on the microstructure.

**Figure 5:**
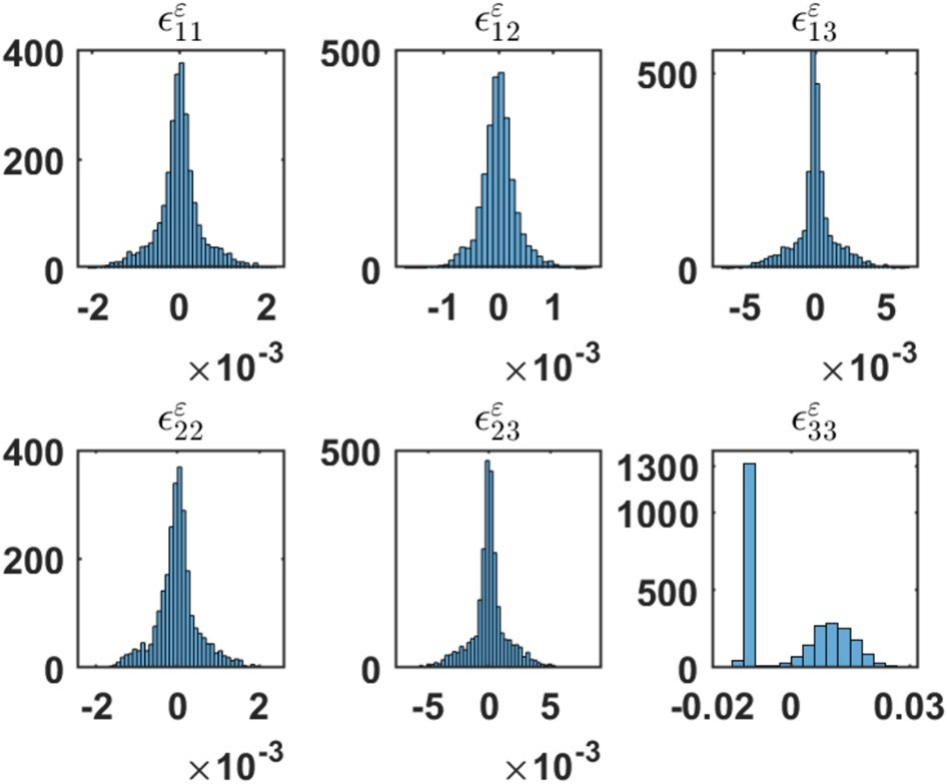
Histogram of the local strain ($\epsilon _{ij}^\varepsilon$) values consequent to the application of external magnetic field (averaged over finite elements). Here one can incisively go into the microstrains spread across the entire composite microstructure.

The results of local mechanical field quantities are summarised in Fig. 4. The magnetic field can permeate the composite structure through the magnetostrictive interaction at the magnetic phase of the composite (i.e., CFO) and then elastically mediated to the ferroelectric phase (STO). The anisotropic magnetostriction exhibited by CFO is very critical in imparting ME coupling in this polycrystalline setting^35,36^. The ensuing microscopic elastic strain (*local strains*) build-up is investigated. The relatively staggered strains/stresses in the FM phase in Fig. 4 can be deducible from the polycrystalline nature of CFO. Noticeable areas of microstructure with grains preferentially oriented according to the magnetic field dot CFO polycrystal. Relatively bigger concentration of stress can be visible in the FE phase (see the subplot von Mises stress $\sigma _v^\varepsilon$ in Fig. 3). The magnitude of micro strain tensor components can be discernible from the histograms of local strains at each finite element (every element is a surrogate of crystallographic grains constituting the polycrystalline FE and FM phases.) and is shown in Fig. 5.

### Design of ME composites

Based on the inputs from the simulation of ME composite we have synthesised a laminate composite thick film choosing the volume fraction of FE over FM as 0.5. The simulation also reveals that a certain degree of randomness in orientation incorporated into the FE phase obviously foster local disorder, which can eventually enhance the ME coupling of the composite system. With this aim in mind and to realize a composite where the magnetic and electric domains could be engineered, we developed a layered composite using CFO with Pr doped STO (Pr:STO). Here the doping in the FE phase STO is to introduce local disorder that can introduce piezoelectricity and potentially enhance the ME coupling. In addition to doping, crystal structure, space group, and ionic polarizability, can also influence the polarization intensity. According to previous experiments on Pb-based ferroelectric materials, rare-earth dopants are responsible for changing the ordering degree of B-site cations by the indirect introduction of random fields/bonds^17,20,21^. The all printed ME composite CFO–Pr:STO exhibited a better magnetoelectric response under the application of dc magnetic field with an appreciable magnetoelectric coupling coefficient (MECC) value ($\alpha _{E}$=779 mV/cm Oe). Relevant properties of individual ferroic layers as well as the composite form were studied in detail and is shown in Supplementary material.

### Magnetoelectric properties of Pr doped STO-CFO heterostructures

Cobalt acetate and iron nitrate react to form CFO which is the magnetostrictive constituent of the ME composite. This is evident from the investigation of thermal decomposition of prepared CFO powder using TG/DTA analysis, as shown in Supplementary figure Fig. S1(a). The CFO synthesized is constituted of single phase material as seen in the XRD pattern of post heat treated CFO at 900$^\circ$C (Supplementary figure Fig. S1(b)). All the peaks in the pattern can be indexed to single phase spinel cobalt ferrite structure [JCPDS File Card No. 22-1086]. CFO show ferromagnetism at 300 K, 500 K, 700 K while exhibit a paramagnetic behavior at 800 K (The M-H loop of CFO at different temperatures shown in Supplementary figure Fig. S1(c)). At a temperature range 700 –800 K, the material changes its nature from ferromagnetic to paramagnetic and hence the Curie temperature too lies in between these two temperatures. Also, one can observe a decrease in saturation magnetization ($\textbf{M}_s$) as the temperature is increased from 300 to 700 K. This is possibly due to the rearrangement of cation distribution, i.e., there will be a degree of inversion in CFO wherein an exchange of Co$^{2+}$ and Fe$^{3+}$ from octahedral to tetrahedral sites and *vice versa* can happen when temperature is increased^37^. The rheological behaviour, which is critical for the assessment of the feasibility of the ink for printing of CFO is shown in Supplementary figure Fig. S1(d). The surface SEM was recorded for the printed film and is shown in Supplementary figure Fig. S1(e). The 3D AFM image of the printed pattern after double stroke printing is shown in Supplementary figure Fig. S1(f).

**Figure 6:**
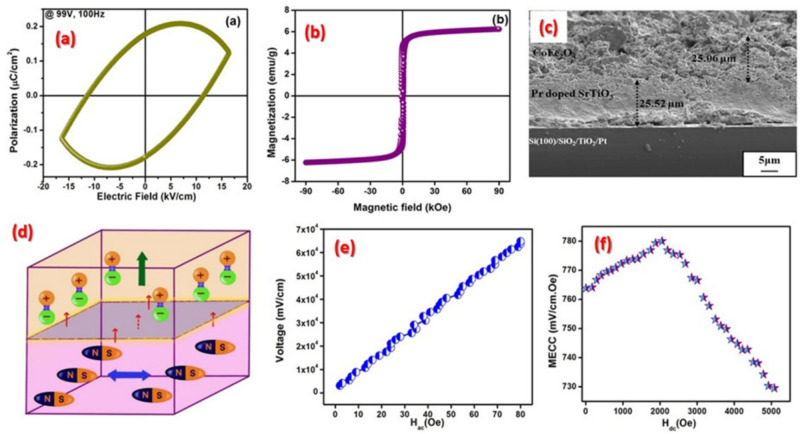
(**a**) P-E loop and (**b**) M-H loop of Pr:STO-CFO bilayer composite at 300 K, (**c**) cross sectional SEM image of Pr:STO and CFO (2-2) composite, (**d**) schematic of ME coupling in Pr.STO-CFO bilayer composite, and variation of ME coupling coefficient under the application of (**e**) DC field and (**f**) AC field for the Pr:STO-CFO bilayer composite at room temperature.

**Figure 7:**
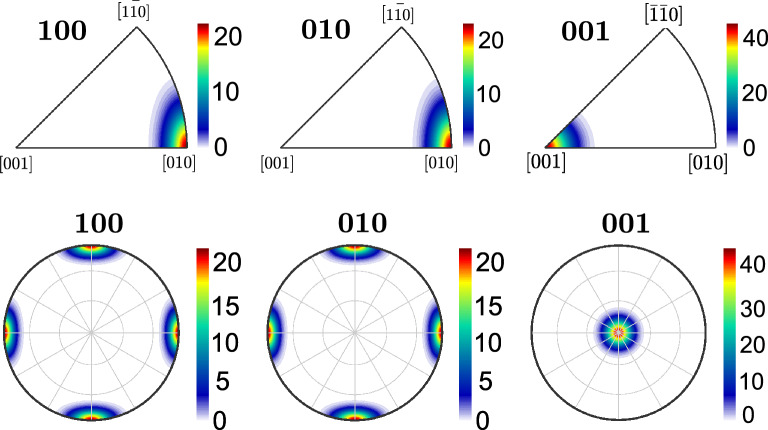
Inverse pole figures of the polycrystalline STO layer, while the orientation distribution parameters are ($\mu _\phi = 0, \sigma _\phi =1,\mu _\theta = 0, \sigma _\theta =1, \mu _\psi = 0, \sigma _\psi =1$). At this configuration, the ME composite delivers maximum $\langle M_3\rangle$ and $\langle P_3\rangle$.

Similarly, Pr doped STO is synthesized using a modified sol-gel synthesis route, which is calcined at 900$^\circ$C. Initially the ink formulated using Pr-STO is screen printed on platinized silicon substrate. This is followed by printing of CFO on top of Pr-STO, resulting in a 2-2 magnetoelectric composite. A leaky dielectric behaviour is observed and is resulted in a lower value of remanence ($P_r$) when compared to Pr:STO printed thick film alone as shown in the P-E curve of Pr:STO-CFO bilayer composite at room temperature in Fig. 6a. This is due to the presence of CFO layer in between the electrodes which is attached to Pr:STO layer. The CFO is clamped with the Pr:STO layer which in turn delimits the electric field to polarize the dipoles close to it. This will result in a lower polarization value as reflected in the FE curve.

The M-H hysteresis curve at room temperature of bilayer composite is shown in Fig. 6b. Even though the FM nature is retained in the composite, $M_s$ value decreased considerably. This can be attributed to the interaction of Pr:STO with CFO. The dipoles in Pr:STO due to the influence of ME coupling at the interface (or due to demagnetization field), will restrict the spins to align in the applied magnetic field which will result in a lower value of $M_s$. The quasi-FE and FM nature present in the composite shows the presence of a spin-charge interaction in the composite that may give rise to ME coupling in the composite.

A complete set of characterization was done for Pr doped STO nanopowder and its quasi-ferroelectric printed pattern. In order to get an idea regarding the ME property of the composite developed with Pr doped STO and CFO, first screen printed one over another on top of a platinized silicon wafer as evident from cross-sectional SEM of the composite shown in Fig. 6c. The SEM image reveals compatibility between the Pr:STO and CFO layers since no visible delamination and cracks between these two layers is observed. The average thickness for the Pr:STO layer is found to be 25.52 $\pm$ 0.03 $\upmu$m and for CFO layer it is 25.06 $\pm$ 0.03 $\upmu$m. Seemingly dense microstructure is also observed in both the layers.

In case of composite ME materials, magnetic and electric terms in the expression for free energy corresponds to ferromagnetic and ferroelectric components. The ME term defines effectual interaction between order parameters for the corresponding phases. This coupling usually originates at the interface between respective phases (see Fig. 6d). This interface breaks symmetry in materials which will further allow the presence of ME coupling in case of composite^38^.

Now examining the peculiar ME coupling of the bilayer laminates, we observe the coupling in ME heterostructures arises due to the strain transfer between the phases. The interface that separates two layers are well defined in this case, which is clear from the cross sectional SEM (see Fig. 6c). When a magnetic field is applied against the bilayer heterostructure, a strain will be generated in the magnetostrictive phase which will further be transferred to the attached FE layer at the interface. So at the output, there will be an electric polarization generated from the FE layer. Hence, the ME coupling in these kinds of layered heterostructures occurs from the elastic interaction between the FE-FM phases.

The ME coupling in the Pr:STO-CFO composite was studied for both ac and dc magnetic fields. The strain induced ME voltage at the output is given by equation (5). Here, for the ME coupling coefficient (MECC) measurement was taken for $\sim$55–60 $\upmu$m thick sample. When ac magnetic field was applied, voltage increases linearly as shown in Fig. 6e. The MECC for this ac response was obtained to be at 780 mV/cm.Oe. The dc response was also recorded for the composite (Fig. 6f). The MECC value increases up to a maximum value at a dc magnetic field and decreases thereafter. The maximum MECC value obtained for dc response in the Pr:STO-CFO bilayer composite is 779 mV/cm.Oe. Since this is a layer-by-layer structure with thickness in the range of microns, the domain walls under the application of external magnetic field will progress much easily when compared to other tape casted laminates. So far as screen print generated lead free ferroelectric-ferromagnetic ceramic composites with 2-2 connectivity are concerned, to the best of our knowledge, there is no reports available dealing with their magnetoelectric coupling. Yet, the present report outperforms the reported literature on bi-layer FE-FM all-ceramic laminates prepared through other methods (see Supplementary material Table S2).

### Homogenized values

**Table 2:** Values of average polarization and magnetization of STO–CFO composite.

Method	P$_r(\upmu C/cm^2)$	M$_r(emu/g)$
Simulation	0.2164	1.72
Experiment	0.1767	4.69

Stereographic projection analyses were conducted for graphical representation of orientation of ferroelectric STO matrix of the ME composite. Inverse pole figures (in Fig. 7 ) show the *hkl* directions of the STO crystallites with respect to the crystallographic axes. A clustering of the poles can be seen in the (001) pole figure (subplots on the right panels of Fig. 7 ), indicating dense alignment of (001) poles along the $y_3$-axis of the microstructure. The $\langle 001\rangle$ axes of the STO are where the spontaneous polarization is oriented and thus the direction along which the maximum piezoelectric strain occurs. $\langle 100\rangle$ and $\langle 010\rangle$ axes too display some pole density though not intense as $\langle 001\rangle$ axes. This indicates that the STO crystallites are mostly aligned along the $\langle 001\rangle$ axes at the optimal orientation distribution as in this study.

We have computed the distribution of the local fields upon application of magnetic field as well in this study and computed the average polarization ($\langle \mathrm {P_3}\rangle$) and magnetization ($\langle \mathrm {M_3}\rangle$) of the composite. The values are found to be $\langle \mathrm {P_3}\rangle = 0.2164~\upmu C/cm^2$ and $\langle \mathrm {M_3}\rangle = 1.72~emu/g$, which is comparable to the range of experimental remanent values denoted as $P_r$ and $M_r$ respectively (Table 2). Here it must be noted that the value of $\langle \mathrm {M_3}\rangle$ deviates from the experiment over a factor of two. Here the magnetization and polarization components are along the *z*–axis (equivalent to $y_3$–axis of the microstructure) of the ME composite.

## Discussion

The microscopic fields (or local fields) pertaining to the electrical, magnetic, and spatial degrees of freedom and their coupling interactions at an arbitrary point in the unit cell can be obtained by the asymptotic analysis^39^. The microscopic fields at an arbitrary point of the unit cell can be determined using the asymptotics of the local fields (Supplementary material). The displacement field $\textbf{u}^{\varepsilon }(\textbf{x})$, the electric potential field $\varphi ^{\varepsilon }(\textbf{x})$ and the magnetic potential field $\psi ^\varepsilon (\textbf{x})$, the local strain $\epsilon _{ij}^\varepsilon (\textbf{x})$, electric field $E_j^\varepsilon (\textbf{x})$ and the magnetic field $H_j^\varepsilon (\textbf{x})$ involving details of the microstructure are obtained once the homogenized macroscopic problem is solved.

First we have run the homogenization to evaluate the equilibrium effective properties of the composite to discern the magnetoelectric coupling. An orientation distribution is introduced for the STO layer of the SFO–CFO laminate to render it polycrystalline nature with a texture (see Supplementary material). The grains constituting the polycrystal are assumed to be single crystalline and each of them are assigned with an orientation picked up randomly from a normal distribution.

The magnetic field distribution along the inplane directions of the composite is not as strong as it is in the out-of-plane direction ($H_1^\varepsilon$ and $H_2^\varepsilon$ depicted in Fig. 2). However, we could observe that the microstructure is dotted with magnetizations of antiparallel polarity throughout. Local field distribution analysis guided by homogenization thus enables to reveal the direction of the local magnetization in the STO-CFO composite. CFO possesses a negative longitudinal magnetostriction ($\lambda _{\parallel }< 0$) and thus should produce compressive stresses across that layer of the ME composite against external magnetic field^35^. This is conspicuously expressed and the contention is corroborated as sketched in the subplot $\sigma _{33}^{\varepsilon }$. One can see negative longitudinal stress being generated in response to external magnetic field in the FM (CFO) phase and is being induced/transported to the FE phase (STO) and consequent strain (contractual, as expected) $\epsilon _{33}^{\varepsilon }$ in FE phase. Longitudinal ($\sigma _{33}^{\varepsilon }$) and transverse ($\sigma _{11}^{\varepsilon }$ and $\sigma _{22}^{\varepsilon }$) components of microstress are compressive in nature leaving the room for expansive ones across other directions (characterised by the non-diagonal components of $\sigma _{ij}^{\varepsilon }$) to preserve the volume of the composite microstructure. The shear components of stress display this trend as is shown in Fig. 4.

Asymmetric expansion/compression can be visible in the micro strain profile (Fig. 5). The local strain component $\epsilon _{33}^{\varepsilon }$ intriguingly display compressive nature all across the STO phase. This is in some way counterbalanced by the mostly positive values of $\epsilon _{33}^{\varepsilon }$ across the FM layer of the composite. This entire local elastic response stems from the magnetostrictive response from the CFO layer of the ME composite and the consequent migration of the same to the FE phase through stress/strain mediation. More than an order of magnitude bigger and compressive are majority of elemental longitudinal strains $\epsilon _{33}^{\varepsilon }$. Since the magnetic CFO phase essentially strains contractually (negative strains) against external magnetic field as dictated by the negative magnetostriction ($\lambda _{\parallel }<0$) is validated here^35^. That the $\epsilon _{33}^{\varepsilon }$ panel in Fig. 5 shows clear indication of negative strains largely and substantially outweigh the positive ones. These findings ought to be viewed against the corresponding results obtained for local strains in the single crystal composite where the longitudinal component of the strain, $\epsilon _{33}^{\varepsilon }$ shows no bias (see the Supplementary Fig. S9) in contrast to that in Fig.  5. Thus heterogeneity plausibly plays a crucial role in the enhancement of ME coupling stemming from the non-uniform local strains. The shear components of strain/stress play a decisive role in the precipitation of corresponding piezoelectric coefficient components in the FE phase and the consequent ME coupling^36^. Our computation of the average out-of-plane stress ($\langle \sigma _{33}\rangle = -3.6\times 10^{9}$N/m$^2$), which is negative and hence compressive reinforces the above.

## Conclusions

Computational and experimental analyses on the magnetoelectric composite reveal magnetization regions in magnetoelectric composite. The distribution of microscopic fields in a magnetoelectric composite of 2–2 connectivity due to the application of external electric and magnetic fields. Macroscopic averages of the resulting, elastic, electric and magnetic responses (i.e., mechanical stress, electric displacement and magnetic flux density) are computed from the knowledge of homogenized macroscopic degrees of freedom. A homogenization method combined with a variational formulation is developed for a periodic multiferroic magnetoelectric composite of lowest crystallographic symmetry of the constituent phases through a two-scale asymptotic analysis of the microscopic electric, magnetic and mechanical fields to characterise the impact on each of them of an external field. The methodology is implemented numerically by modifying the software POSTMAT (*material postprocessing*). The ferri/ferromagnetic CFO laminated onto a ferroelectric STO layer is chosen as a typical magnetoelectric composite for simulation. The microscopic fields (or *local fields*) pertaining to the electrical, magnetic, and spatial degrees of freedom and their coupling interactions at any arbitrary point in the unit cell are obtained. A convergence analysis of magnetoelectric properties with unit cell size allows us to determine the simulation-space independent, equivalent magnetoelectric properties of the composite.

Polar magnetic regions (PMRs) (due to external electric field) that are potential locations for information storage in the microstructure are identified in the CFO–STO system. Antiparallel local magnetic field vectors $H_j^\varepsilon$ percolates the microstructure of the composite. We observed longitudinal contraction along the out-of-plane direction ($y_3$ axis) of the composite under external magnetic field from the observation of compressive local strains $\epsilon ^\varepsilon _{33}$ and the negative average out-of-plane stress ($\langle \sigma _{33}\rangle$= -3.6$\times 10^{9}$N/m$^2$). The local strain distribution can be discernible from the histograms of local strains at each crystallographic grain as is shown in Fig. 5. $\epsilon _{33}^{\varepsilon }$ which is the local strain along the $y_3$ axis (which is the out-of-plane direction of the composite) is found to be more than an order of magnitude bigger and compressive compared to other components. This corroborates the negative magnetostriction ($\lambda _{\parallel }<0$) observed previously^35^ in magnetic CFO phase and that can essentially strains contractually (negative strains) the entire composite against external magnetic field is validated. Thus the $\epsilon _{33}^{\varepsilon }$ panel in Fig. 5 shows negative strains largely and substantially outweigh the positive ones. The present computation of the (negative) average out-of-plane stress ($\langle \sigma _{33}\rangle$= -3.6$\times 10^{9}$N/m$^2$), reinforces the compression of the microstructure along the out-of-plane direction.

Another parameter of importance is the overall ME voltage coefficient $\alpha _{E} =\delta E/\delta H$ of the laminate. Generally, $\alpha _{E}$ is determined when the sample is subjected to a bias field $\varvec{H}$ and an AC field $\delta H$ by measuring the electric field $\delta E$^30^. An electric field ($\mathbb {E}$ parallel to $y_3$–axis) sufficient to saturate the material electrically is first applied to the material and the resulting local fields are mapped alongwith the averages. Electric-to-magnetic energy conversion can plausibly be occurred through the transmission of an electric field induced elastic stress from the ferroelectric phase to the magnetic phase. Significant impact of coupling between the magnetic and electric degrees of freedom in the composite is obtained from this analysis. Average magnetization $\langle \mathrm {M_k}\rangle$, is computed ($\langle \mathrm {M_3}\rangle = 1.72~emu/g$) and found to be within reasonable range measured in similar systems. The magnetization $\langle \mathrm {M_3}\rangle$ is found to be along the direction of the applied field which is the out-of-plane direction of the ME composite.

Strong magnetoelectric coupling can be manifested due to an applied magnetic field ($\mathbb {H}$ parallel to $y_3$–axis) as well by the confluence of magnetostrictive effects and piezoelectricity to achieve an electric response. There is an interplay of piezomagnetism coupled with piezoelectricity for this kind of phenomenon to occur in CFO–STO composite. The elastic deformation is generated by magnetostriction of the FM phase and the consequent strain is permeated to the FE material, resulting in an induced electric polarization due to piezoelectric effect. Histograms of local strains mapped in order to help go incisively into the composite microstructure is in conformity with the negative magnetostriction in CFO layer. Average polarization $\langle \mathrm {P_3}\rangle$ resulted from external magnetic field is computed ($\langle \mathrm {P_3}\rangle = 0.2164~\upmu C/cm^2$) manifesting strong cross coupling of electricity and magnetism. This value is in reasonable range of the measured remanent polarization $P_r$ (Table 2). The insights obtained from the present study will provide a basis for extending the application of systematic design procedures in finely resolved scales to new and challenging materials for real-world electronic applications.

## Methods

### CoFe$_2$O$_4$–-Pr:SrTiO$_3$ thick film preparation

Cobalt acetate (Co(CH$$_3$$CO$$_2)_2$$.4H$$_2$$O, Sigma Aldrich), Iron nitrate (Fe(NO$$_3)_3$$.9H$$_2$$O, Sigma Aldrich), Titanium isopropoxide (C$$_{12}$$H$$_{28}$$O$$_4$$Ti, Sigma Aldrich), Praseodymium nitrate hexahydrate (Pr(NO$$_3)_3$$.6H$$_2$$O), Acetyl acetone (C$$_5$$H$$_8$$O$$_2$$, Sigma Aldrich), Ethylene glycol (C$$_2$$H$$_6$$O$$_2$$, Sigma Aldrich), Strontium acetate (C$$_4$$H$$_6$$O$$_4$$Sr, Sigma Aldrich), 2-Methoxy ethanol (2-MOE, Sigma Aldrich) and Diethanolamine (DEA, Sigma Aldrich) were used as the starting materials.

About 0.02 mol of (Co(CH$_3$CO$_2$)$_2$.4H$_2$O and 0.01 mol of (Fe(NO$_3$)$_3$.9H$_2$O were mixed in 2-MOE and DEA in a round bottomed flask, using an ultra sonicator. Vigorous sonication was performed for 2 h till a uniform mixture of precursor was obtained. This solution was kept in an oil bath at 70$^\circ$C and continued heating for 12 h under constant stirring with refluxing in order to avoid evaporation of the solvents. After 12 h, the solution was allowed to dry. The dried powder was then taken for further characterizations.

The Pr doped strontium titanate was synthesized using a sol-gel synthesis route. For this, C$$_{12}$$H$$_{28}$$O$$_4$$Ti was added to C$$_5$$H$$_8$$O$$_2$$ in a round bottom (RB) flask at 120$$^\circ$$C under constant stirring. After 30 minutes C$$_2$$H$$_6$$O$$_2$$ was added to the solution. At the same time, in another RB, sufficient quantity of C$$_4$$H$$_6$$O$$_4$$Sr and Pr(NO$$_3)_3$$.6H$$_2$$O (Sr:Pr= 0.925:0.075) were dissolved in ethylene glycol, C$$_2$$H$$_6$$O$$_2$$, and were kept at 120$$^\circ$$C kept under constant stirring. After 1 h of mixing, both these solutions were mixed together and allowed to stir at 120$$^\circ$$C. After 4 h of mixing, 3 ml deionized water was added to the above mixture to form the stable solution of Pr:STO. The solution was then air dried at 100$$^\circ$$C and the obtained powder was ground well, calcined and qualified for ink preparation.

After the phase formation of Pr:STO and CFO were confirmed and powders were qualified, our next aim was to develop a viscous ink out of the developed powder. The solvents chosen here is a binary solvent system consisting of xylene and ethanol, Triton X-100 as dispersant and ethyl cellulose as binder. A screen printable ink usually consists of filler, solvent, binder as well as dispersant. Here, the fillers are the perovskite oxides Pr:STO and CFO. Binary solutions having equal volumes of xylene and ethanol were chosen as solvents in order to facilitate fast curing for the proposed inks.

(i) In the first step, Triton X-100, the dispersant, was dissolved in solvent mixture for 30 min in an ultrasonic bath.
(ii) Respective fillers (Pr:STO and CFO) was added to the solution and stirred continuously for 4 h in a magnetic stirrer.
(iii) Ethyl cellulose (binder) was added and stirred for another 24 h to get a homogenous ink with desired viscosity.

For the production of hybrid ME composites, these inks were screen printed on desired substrates (preferably, Si(100)/SiO$_2$/TiO$_2$/Pt substrates) one over other and was kept at 100$^\circ$C for 24 h in an oven.

### Characterization

TG/DTA was performed using a thermo gravimetric analyzer from room temperature upto 1000$^\circ$C in oxygen atmosphere (TGA/DTA instruments, Shimadzu, Japan). Phase purity of calcined powder was confirmed using X-ray diffraction technique with $Cu K\alpha$ radiation (X’Pert PRO diffractometer, PANalytical, Almelo, The Netherlands). High resolution TEM was used for analyzing the particle size of developed CFO powder (FEI Tecnai G2 30S-TWIN, FEI Co., Hillsboro, OR, USA). The rheological behavior of the ink was done using Rheo plus32 rheometer (Anton Paar, Ashland, VA, USA). The screen printing of ink on different substrates were performed using XPRT2 semiautomatic screen-printer (EKRA, Germany). Scanning electron microscopy was used for the microstructural analysis of printed films using JSM 5600LV model (JEOL, Tokyo, Japan). Surface roughness of thick film was estimated with the help of atomic force microscopy (AFM) in tapping mode (Multimode, Bruker, Germany). Magnetic measurements were performed using an oven setup attached to the physical property measurement system (PPMS, Quantum Design, USA). ME measurements were carried out in ac as well as dc magnetic fields. The ac magnetic field was applied with the help of a pair of Helmholtz coils using which, a suitable magnetic field was applied across the sample and the ME output voltage was estimated by recording the induced polarization (P). The working principle is that the electric dipoles inside the sample align themselves with the ac magnetic field, resulting a drop in ac voltage across the sample which is measured via Faraday Effect. Thus it is clear that the apparent output voltage is purely due to the ME coupling effect. For measuring the dc field dependence of ME coupling, a setup consisting of dc electromagnet having bipolar power supply with GPIB integrated gauss meter. Both these fields were applied along the direction of thickness of the printed heterostructure. The data acquisition was performed using LabVIEW software. Now, the electric as well as magnetic inductions in a ME laminate can be expressed as,

$$\begin{aligned} P_i=\dfrac{\partial F}{\partial E_{0i}}&=P_{Si} +\epsilon _0 \sum _j\chi _{ij}^e E_{0j} +\dfrac{1}{\mu _0}\sum _j\alpha _{ij} B_{0j}+\cdots \\ M_i=\dfrac{\partial F}{\partial B_{0i}}&=M_{Si} +\dfrac{1}{\mu _0}\sum _j\chi _{ij}^m B_{0j} +\dfrac{1}{\mu _0}\sum _j\alpha _{ij} E_{0j}+\cdots \end{aligned}$$

From these two equations, it is clear that, there is a component for cross polarization irrespective of spontaneous and induced terms. The linear ME effect is given by, $\alpha _{ij}=\dfrac{\partial P_i }{\partial B_{0j}}= \mu _0 \dfrac{\partial M_i}{\partial E_{0j}}$ For an isotropic material, $\alpha ^2=\epsilon _0\mu _0 \chi ^e \chi ^m$. This suggests that, large ME coupling can be observed in materials with large electric and magnetic susceptibility values.

### Simulation using homogenization

The equivalent material properties of a periodic multiferroic magnetoelectric composite at equilibrium are determined using a two-scale asymptotic homogenization analysis^29^. The equivalent ME coupling thus obtained is given by

2 $$\begin{aligned} & \widetilde{\alpha }_{im}= \frac{1}{\left| Y \right| }\int _Y \Big [ e_{pkl}(\textbf{x,y}) \frac{\partial \Gamma ^{m}_k}{\partial y_l}- \kappa ^{\epsilon H}_{pj}(\textbf{x,y})\frac{\partial \Psi ^{m}}{\partial y_j} \nonumber \\ & -\alpha _{pj}(\textbf{x,y})\Big (\delta _{jm}+ \frac{\partial Q^{m}}{\partial y_{j}} \Big )\Big ] \nonumber \\ & \times \Big (\delta _{ip}+\frac{\partial R^i}{\partial y_p}\Big )\mathrm d Y \end{aligned}$$

Here *R* and *Q* are characteristic electric and magnetic displacements. $\mathbf {\Gamma },$ and $\Psi$ are characteristic coupled functions satisfying a set of microscopic equations^29^. These characteristic functions appear in the microscopic displacement, electric and magnetic field perturbations, *viz.*, $\mathbf {u^1(x,y)}$, $\varphi ^1\mathbf {(x,y)}$ and $\psi ^1\mathbf {(x,y)}$ respectively (given in Supplementary material), due to the microstructure heterogeneity. The spacial derivatives of the macroscopic displacement, electric and magnetic fields *viz.*, $\mathbf {u^0(x,y)}$, $\varphi ^0\mathbf {(x,y)}$ and $\psi ^0\mathbf {(x,y)}$ can bridge the macroscale and the microscale through the characteristic functions mentioned above. $e_{pkl}$ and $\kappa _{pj}^{\epsilon H}$ are the piezoelectric coefficient and dielectric permittivity at constant strain $\epsilon$ and magnetic field vector $\textbf{H}$ respectively. $i, j, k, l, \dots = 1,2,3$ are the 3–dimensional coordinate indices, and $\delta _{ij}$ are the Kronecker delta symbol. Here we assume Einstein convention on summation about repeated indices. (Here the $\sim$ over Greek or Latin letters represents homogenized values). We consider the ME composite as a heterogeneous body obtained by the translation of microstructure of size *Y*. For the homogenized ME composite laminate, the physical properties do not depend on $\textbf{x}$, the global frame of reference. Instead, if the material is heterogeneous, the magneto-electro-mechanical properties effectively depend on $\textbf{x}$ such that $\mathbf \kappa ^{\epsilon H} \equiv \mathbf \kappa ^{\epsilon H}(\textbf{x}), ~ \textbf{e}\equiv \textbf{e}(\textbf{x}), \dots$ etc. The material properties $\mathbf \kappa ^{\epsilon H}$, $\textbf{e}$, $\mathbf \alpha$, etc as well as the characteristic functions $R, Q, \mathbf {\Gamma }$, and $\Psi$ are *Y*–periodic functions depending on the microscopic coordinates $\textbf{y}$.

The equivalence of the average stress and homogenised stress can easily be expressed by applying the average operator $\langle \centerdot \rangle$ denoting $(\frac{1}{\left| Y \right| }\int _Y \centerdot dY)$, where *Y* is the domain size. Hence the average fields are computed to be

3 $$\begin{aligned} \langle \sigma _{ij}\rangle= & \widetilde{C}^{EH}_{ijkl}\Bigg (\frac{\partial u^0_k(\textbf{x})}{\partial x_l}\Bigg ) +\widetilde{e}_{kij}\Bigg (\frac{\partial \varphi ^0(\textbf{x})}{\partial x_k}\Bigg )+\widetilde{e}^{~M}_{kij} \Bigg (\frac{\partial \psi ^0(\textbf{x})}{\partial x_k}\Bigg ) \end{aligned}$$

4 $$\begin{aligned} \langle D_{i}\rangle= & \widetilde{e}_{ijk}\Bigg (\frac{\partial u^0_j(\textbf{x})}{\partial x_k}\Bigg )- \widetilde{\kappa }^{\epsilon H}_{ij}\Bigg (\frac{\partial \varphi ^0(\textbf{x})}{\partial x_j}\Bigg )-\widetilde{\alpha }_{ij}\Bigg (\frac{\partial \psi ^0(\textbf{x})}{\partial x_j}\Bigg ) \end{aligned}$$

5 $$\begin{aligned} \langle B_{i}\rangle= & \widetilde{e}^{~M}_{ijk}\Bigg (\frac{\partial u^0_j(\textbf{x})}{\partial x_k}\Bigg )- \widetilde{\alpha }_{ji}\Bigg (\frac{\partial \varphi ^0(\textbf{x})}{\partial x_j}\Bigg )-\widetilde{\mu }^{\epsilon E}_{ij} \Bigg (\frac{\partial \psi ^0(\textbf{x})}{\partial x_j}\Bigg ) \end{aligned}$$

These *macroscopic equations* (i.e. they do not contain $\textrm{y}$) can be computed once the homogenized solution for $\textbf{u}^0$, $\varphi ^0$ and $\psi ^0$ and that of the corresponding fields *viz.,* $\frac{\partial u^0_j(\textbf{x})}{\partial x_k}$, $\frac{\partial \varphi ^0(\textbf{x})}{\partial x_j}$ and $\frac{\partial \psi ^0(\textbf{x})}{\partial x_j}$ are prescribed. This *postulate* is equally applicable for the case with the microscopic fields and for the microscopic stress, electrical displacement and flux densities.

Both the constituents of the system of 2–2 magnetoelectric composite consisting of layer of FE material (STO) glued into a laminae with a layer of FM material (CFO) are of equal volume. We use, single crystalline data for STO (from Ref. ^32^) and polycrystalline data for CFO (from Ref. ^40^).

The numerical implementation is based on the software POSTMAT(*material postprocessing*) developed by Guedes and Kikuchi^39^, which is explained in Supplementary material.

## Supplementary Information

Supplementary Information.

## Data Availability

The datasets generated during and/or analysed during the current study are available from the corresponding author on reasonable request.
