# Supplementary material for: Magnetic and electric polar regions in the magnetoelectric composite microstructure
Source: Sci Rep. 2025 Oct 22;15:36904. doi: 10.1038/s41598-025-20911-z (PMC12546733; doi:10.1038/s41598-025-20911-z)
Supplement: Supplementary file 1 — Supplementary Information. [file 41598_2025_20911_MOESM1_ESM.pdf]

Supplementary material to Magnetic and electric polar regions in the magnetoelectric composite microstructure

K. P. Jayachandran,[1,2] Deepa Rajendran Lekshmi,[3] J.M. Guedes,[1] K.P. Surendran,[3] and H.C. Rodrigues[1]

[1)] *IDMEC, Iinstituto Superior Técnico, Universidade de Lisboa, Av. Rovisco Pais, 1049-001 Lisboa, Portugal*

[2)] *School of Pure and Applied Physics, Mahatma Gandhi University, Kottayam 686560, India*[a)]

[3)] *Materials Science and Technology Division, CSIR- National Institute for Interdisciplinary Science and Technology Division (CSIR-NIIST), Thiruvananthapuram 695019, India*

Using a combination of continuum simulations, atomic force microscopy, physical property measurement system and screen printing of bilayer magnetoelectric composite on conductive substrates we demonstrate robust room-temperature magnetoelectric coupling in rare earth substituted $SrTiO_3$–$CoFe_2O_4$ system.

[a)]Electronic mail: kpjayachandran@gmail.com

I. PROPERTIES OF CFO POWDER

The thermal decomposition of prepared CFO powder was investigated using TG/DTA analysis, as shown in figure S1(a). Two apparent weight losses are observed in the TG profile, one minor and the other major. First one is a weight loss of around 5.4%, observed upto 100°C. The prominent weight loss of around 51% is observed between 150°C and 350°C for which a sharp exothermic peak at 313°C in the DTA curve. The XRD pattern of post heat treated CFO at 900°C is shown in figure S1(b). All the peaks in the pattern can be indexed to single phase spinel cobalt ferrite structure [JCPDS File Card No. 22-1086]. No intermediate or additional peaks can be observed in the obtained pattern within the sensitivity of experimental technique. The M-H loop of CFO at different temperatures were recorded and shown in figure S1(c). This reveals that the material show ferromagnetism at 300 K, 500 K, 700 K while exhibit a paramagnetic behaviour at 800 K. So at some temperature between 700 K and 800 K, the material changes its nature from ferromagnetic to paramagnetic behaviour and hence Curie temperature lies in between these two temperatures. Also, one can observe a decrease in saturation magnetization (M_S) when temperature is increased from 300 K to 700 K. This is possibly due to the rearrangement of cation distribution, i.e., there will be a degree of inversion in CFO wherein an exchange of Co^{2+} and Fe^{3+} from octahedral to tetrahedral sites and vice versa can happen when temperature is increased .

II. PROPERTIES OF Pr DOPED $SrTiO_3$

X-ray diffraction was used in the present study as a primary tool to check the phase purity of ferroic materials since the presence of any inorganic impurity or additional phase in the compound may drastically affect their performance. The XRD pattern of Pr:STO was recorded, as shown in S2(a). The precursor powder was calcined from 500°C to 900°C for 4 h in a muffle furnace. The resultant XRD patterns indicate that the phase formation begins from 600°C and is completed at 900°C. All the peaks in the XRD pattern can be indexed using the standard JCPDS file 89-4934. Interestingly, the crystal structure belongs to I4/mcm space group with tetragonal symmetry (which can be ferroelectric), in sharp contrast to

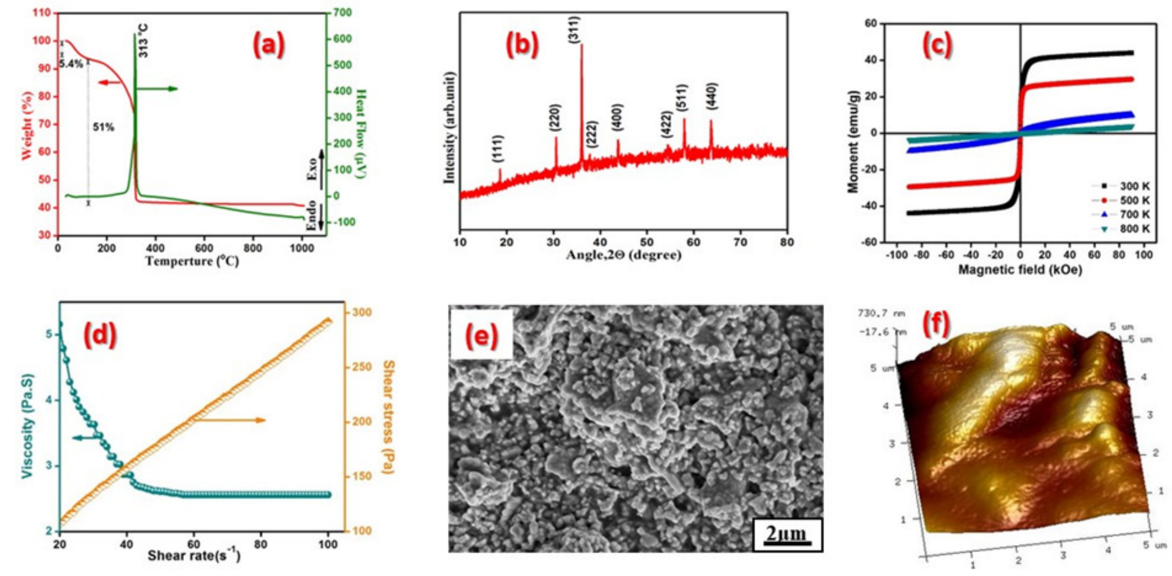

Figure S1. (a) TG/DTA profile and (b) XRD of as synthesized CFO powder, (c) M-H curves of CFO at different temperatures, (d) variation of viscosity and shear stress of CFO ink with shear rate, (e) SEM micrograph of printed CFO surface and (f) 3D AFM image of double stroke screen printed CFO

usual cubic STO phase which is paraelectric. The sharpness of the diffraction peaks indicates its better homogeneity and crystalline nature. In order to understand the thermal evolution history of the precursor powder of Pr:STO, a combined TG/DTA was performed, whose results in terms of weight loss and heat flow as a function of temperature. The TG curve was recorded for the STO sample in oxygen atmosphere with 5°C/min heating rate from 30-1000°C which is shown in Fig. S2(b). As suggested by the TG curve, thermal decomposition profile of STO can be divided into four stages. In the first stage, the weight loss starts from 30°C and shows a sharp decrease up to 100°C indicating a total weight loss of around 6.66%. This can be attributed to the removal of planar water and ethylene glycol present in the respective powder. In DTA curve, an endothermic peak is observed around 56°C, corresponds to the foresaid weight loss. In the following stage, a steady weight loss of about 5.33% is observed up to 260°C. Major weight loss of around 24% is observed after 260 to 400°C. This may be due to the removal of volatile organic residues present in the powder sample. The sharp exothermic peak at around 326°C corresponds to the removal of alkoxides and acetates which were added as reagents during the reaction process. A TG weight loss of about 8.3%

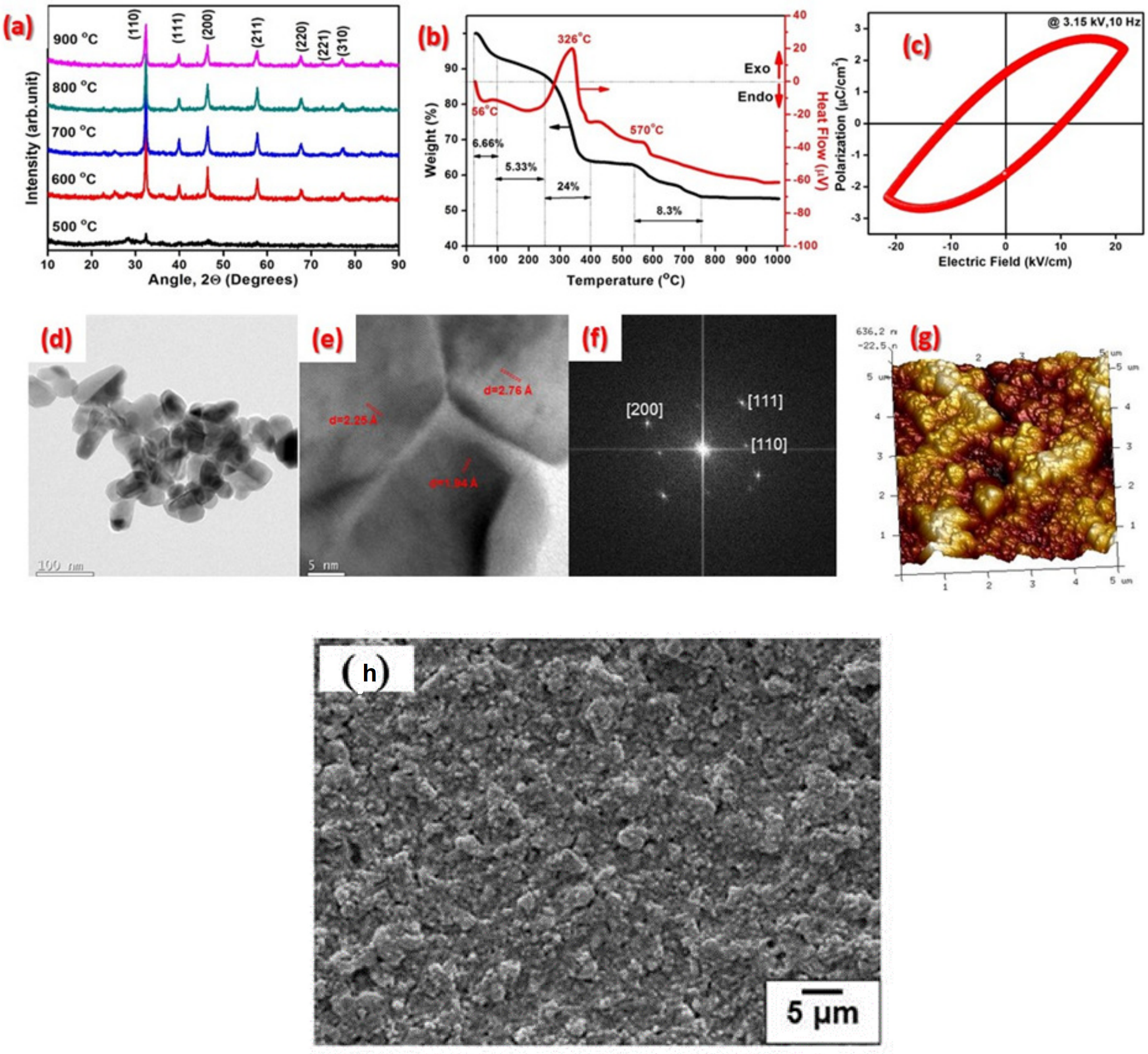

Figure S2. (a) XRD patterns, (b) TG/DTA analysis, (c) Room temperature P-E hysteresis curve, (d) , (e) and (f) HRTEM images and FFT pattern and (g) 3D AFM images (h) SEM micrograph of Pr:$SrTiO_3$, screen printed on platinized silicon substrate.

is observed from 550 to 750°C and thereafter no considerable weight loss is observed. The loss of weight in this particular region is implied to the burn out of surface hydroxyls and carbon residues. An exothermic peak around 570°C is attributed to this weight loss. Based on this TG-DTA result, the prepared powder was calcined over a temperature range from 500°C to 900°C. The XRD pattern of Pr:STO (Pr doped $SrTiO_3$)was recorded, as shown in

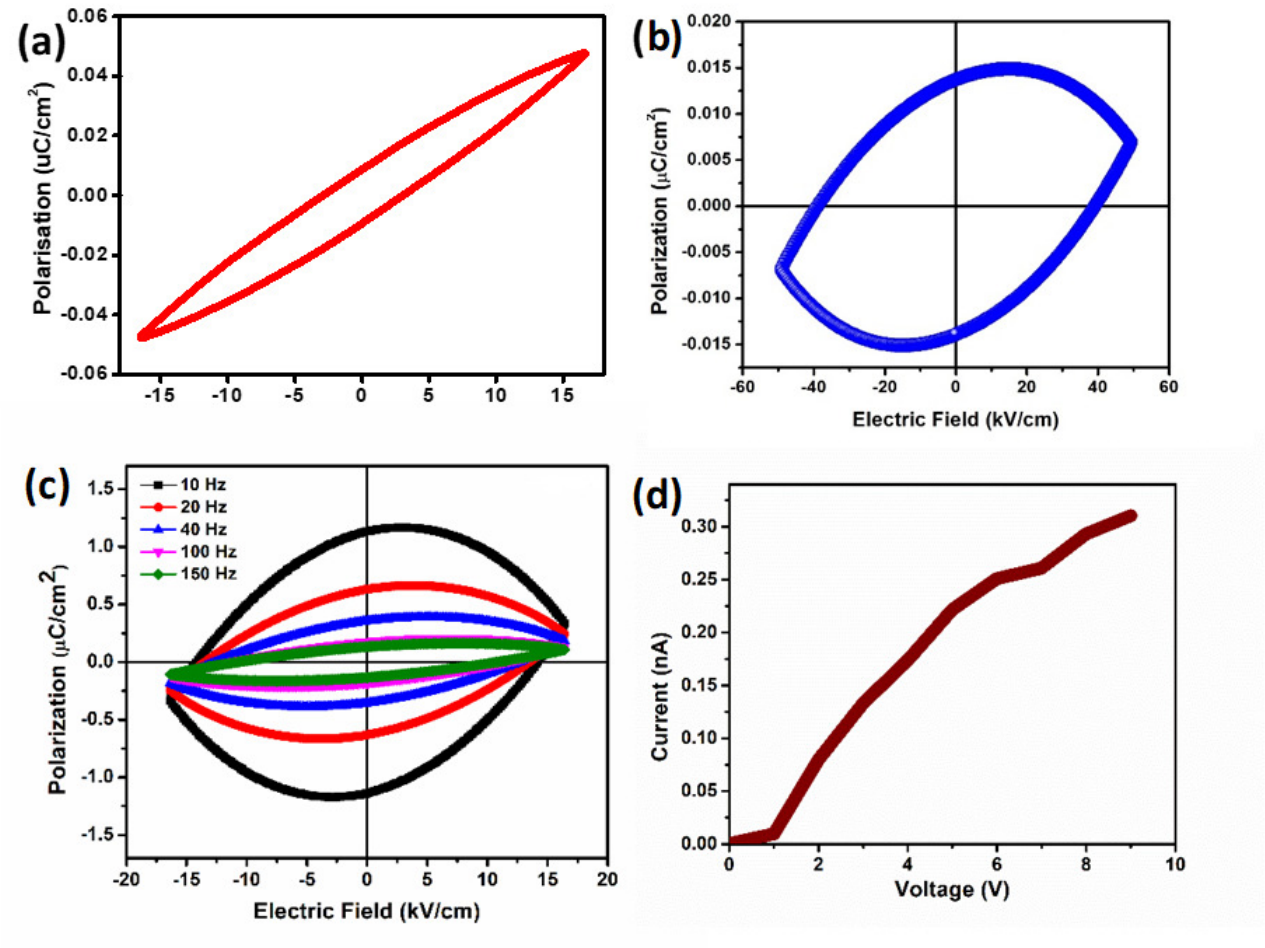

Figure S3. (a) P-E loop response of pure STO and (b) Pr:STO screen printed films. P-E response of (c) Pr:STO-CFO composites at various frequencies in the range 10 Hz-150 Hz, and (d) I-V characteristics of screen printed Pr:STO laminate.

figure Fig. S2(a). The TG curve was recorded for the STO sample in oxygen atmosphere with 5°C/min heating rate from 30°C-1000∘C which is shown in figure Fig. S2(b). Figure Fig. S2(c) shows the room temperature variation of polarization (P) with respect to electric field (E) of STO thick films which were printed on platinized silicon substrates. Figure Fig. S2(d) represents the morphology as well as particle size of calcined STO. The crystalline nature of prepared powder is clear from the lattice image shown in figure Fig. S2(e). The Fast Fourier Transform (FFT) pattern indicated in figure Fig. S2(f) corresponds to six diffraction spots, emanating from the STO crystal planes. Surface morphology of the printed patterns

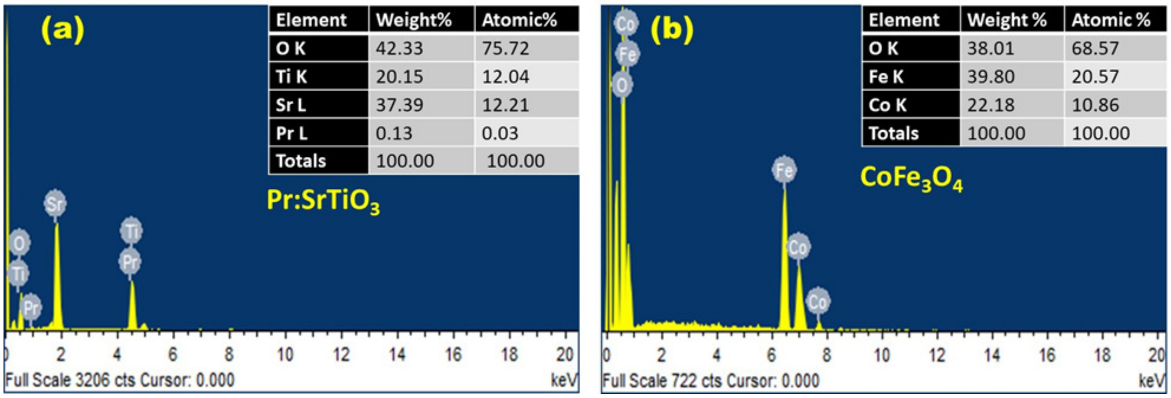

Figure S4. (a) & (b) EDS spectra of screen printed Pr:$SrTiO_3$ and $CoFe_2O_4$ respectively.

as viewed using AFM, is shown in figure Fig. S2(e).

The P-E hysteresis of Pr doped $SrTiO_3$ is now provided. As well known, $SrTiO_3$ is an incipient ferroelectric perovskite and a quantum paraelectric material, in which quantum fluctuations and anti-ferrodistortive instabilities suppress ferroelectric polar order at low temperature, thus resulting in a nonpolar paraelectric state. This is clearly shown in Fig. S3 (a), which is characteristic of a leaky dielectric. However, on doping with Pr^{3+} at $SrTiO_3$ lattice can result compressive chemical pressure in the lattice, leading to a noncentrosymmetric state induced by lattice strain [A. Durán et al., J. Appl. Phys. 97, 104109 (2005)]. Fig. S3(b) shows the P-E hysteresis of Pr doped STO which is screen printed on platinized silicon. Other researchers also have observed peculiar dielectric relaxation with high polarization response to applied electric field in Pr doped $SrTiO_3$ [(i) T. Wei et al., Funct. Mater. Lett., 5 (1), 1–5 (2012), (ii) X. Wang et al., Solid State Commun., 150 (5–6), 267–270 (2010)]. It should be noted that the induced ferroelectricity is weak. As suggested by the reviewers, the I-V characteristics of the STO is also tested which is found to be that of an insulator, as shown in Fig. S3(d). We have also tested the P-E response of the layered 2-2 Pr:STO-CFO composites at various frequencies in the range 10-150 Hz. The introduction of the ferrite seemingly degrades the piezoelectric characteristics of Pr:STO. It is found that as the frequency increases, the 2-2 composite behaves more towards a lossy dielectric which may be due to the influence of the more conductive ferromagnetic CFO phase.

The surface SEM images of STO screen printed pattern and CFO screen printed pattern

are shown in figure Fig. S2(h) and figure Fig. S1(e) respectively. A uniform distribution of particles can be observed throughout the microstructure with minimal porosity. The porosity in screen printed samples is an unavoidable factor. It arises due to the fast volatilization of organic solvents present in the ink formulation. Also, we do not employ post printing sintering for screen printed sample. Hence, there will be porosity in the (2-2) composite made using STO and CFO inks.

As the samples are not heat treated post sintering, the relative density of the printed sample cannot be measured. Nevertheless, one can notice that the average particle size is $< 1\ \mu m$ for both STO and CFO. The EDS spectra of individual films of both Pr:$SrTiO_3$ (STO) and $CoFe_2O_4$ (CFO) thick films were recorded, and a representative spectra is given in Figs. S4(a) and (b). It may be noted that the stoichiometry of $SrTiO_3$ is well maintained with trace amount of praseodymium can also be observed within the limits of experimental error (Fig. S4(a)). The $CoFe_2O_4$ is also appeared to be phase pure with the desired stoichiometry (Fig. S4(b)).

The doping of trivalent rare earth ion can happen in different ways. The first possibility is a partial substitution of Ti^{4+} by trivalent Pr^{3+} maintains charge neutrality by excluding the formation of Sr vacancies. Alternatively, planar faults of the host are compensated by the trivalent impurity, through radiative recombination . Fig. S2(c) shows the room temperature variation of polarization (P) with respect to electric field (E) of STO thick films which were printed on platinized silicon substrates. It can be shown that the switched charge (Q) of a real dielectric is given by[1],

$$Q = 2P_rA + \sigma EAt$$

where P_r is the remanent polarization, A is the area of the capacitor, σ is the electrical conductivity of the real dielectric, $\mathbf{E}$ is the applied field and t is the measurement time.

In the case of a quasi linear dielectric[2],

$$Q = \sigma EAt$$

which means that it has finite leakage and is lossy, since σ is proportional to dielectric

loss ($\tan\delta$). Here the leakage current may be originated from the oxygen vacancies that may likely to form by the partial substitution of Ti^{4+} by Pr^{3+}. The P-E hysteresis loop in the present case indicates that Pr:STO not a perfect ferroelectric, but more like a quasi-ferroelectric or lossy dielectric, even though there were earlier reports indicating the room temperature ferroelectricity in the same. Nevertheless, more research is needed to confirm the 'ferroelectric-like' behaviour of Pr-$SrTiO_3$ at ambient conditions, which is beyond the scope of present work. The Pr-STO sample was able to bear an electric field up to 20 kV/cm without any break down, but it is observed that even with the application of such a high electric field, the P-E loop obtained is not fully saturated, typical of quasi ferroelectrics. The remanent polarization (P_r) and coercive field (E_c) obtained are 1.61 μC/cm2 and 10.3 kV/cm respectively. Beyond 20 kV/cm, the sample undergoes break down.

The nanocrystalline nature of Pr:STO as well as STO was analyzed using TEM analysis. Fig.S2(d) represents the morphology as well as particle size of calcined STO. It is evident from the figure that Pr:STO has an average particle size ranging from 30 nm to 60 nm. The crystalline nature of prepared powder is clear from the lattice image shown in Fig. S2(e). The value of d-spacing between adjacent fringes is measured to be 1.94 Å, 2.25 Å and 2.76 Å which corresponds to [200], [111] and [110] planes respectively. The Fast Fourier Transform (FFT) pattern indicated in Fig. S2(f) corresponds to six diffraction spots, emanating from the STO crystal planes. The corresponding d-spacing values for these reflections were analyzed and they were originated from [200], [111] and [110] planes of respective crystal.

Surface morphology of the printed patterns as viewed using AFM, is shown in Fig. S2(e). Surface smoothness plays an important role in determining the quality and adhesion of the printed pattern. As the solvents in used in ink formulation escapes at room temperature, there includes some porosity in the dried printed patterns we developed. AFM micrographs were recorded from the surface of double stroke printed samples. Fig. S2(e) represents 3D image of STO samples. The average surface roughness (R_a) and root mean square roughness (R_q) of STO samples were analyzed to be 91.7±5 nm and 117±5 nm respectively. Two important parameters obtained from AFM images are kurtosis topography and skewness. In the present case for STO, kurtosis value is observed to be 2.72. Usually kurtosis value < 3 indicates that the distribution curve is platykurtoic, which means that scan surface consists

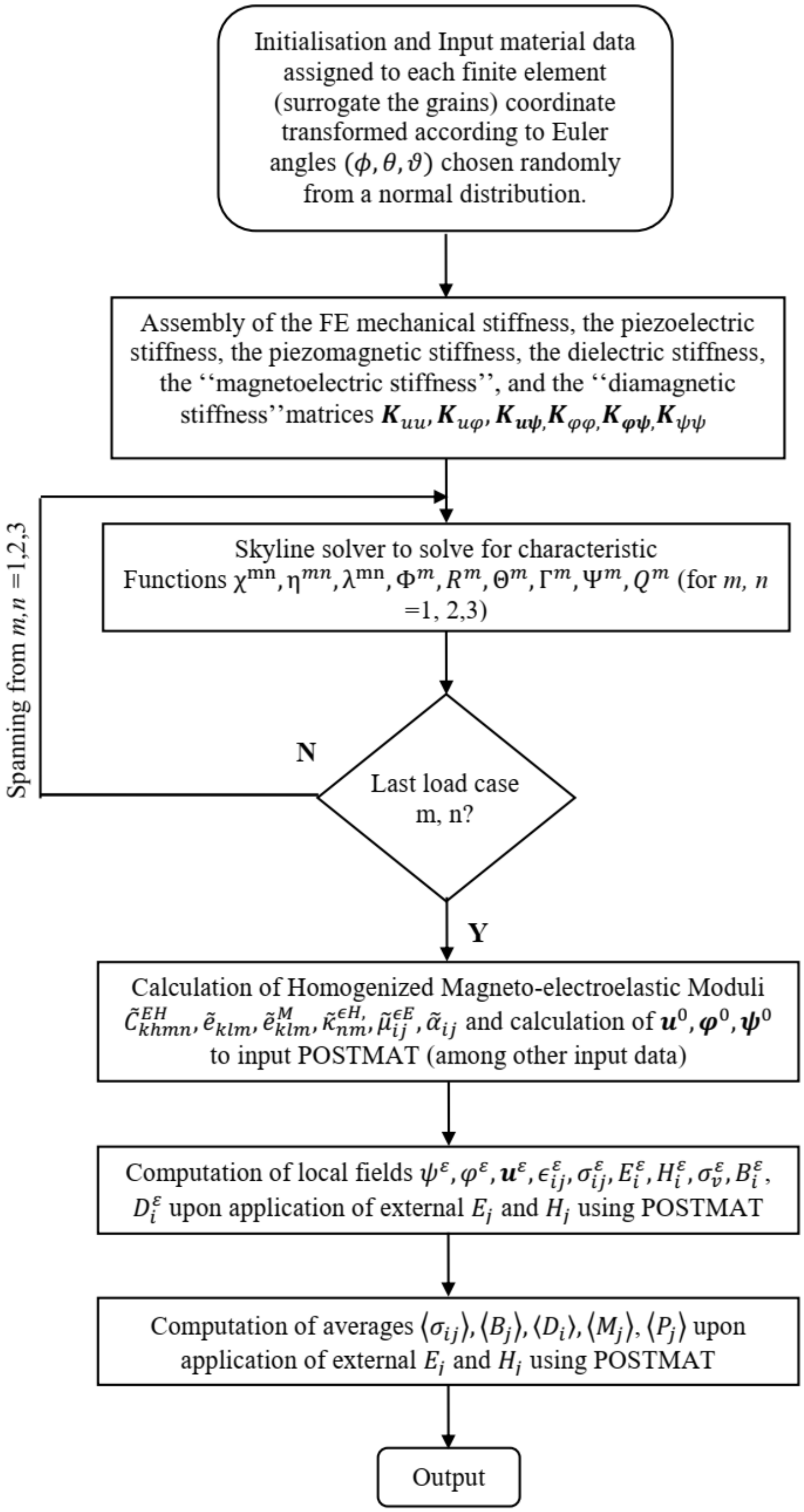

Figure S5. Workflow showing the homogenization procedure and the POSTMAT for computing local fields and average fields.

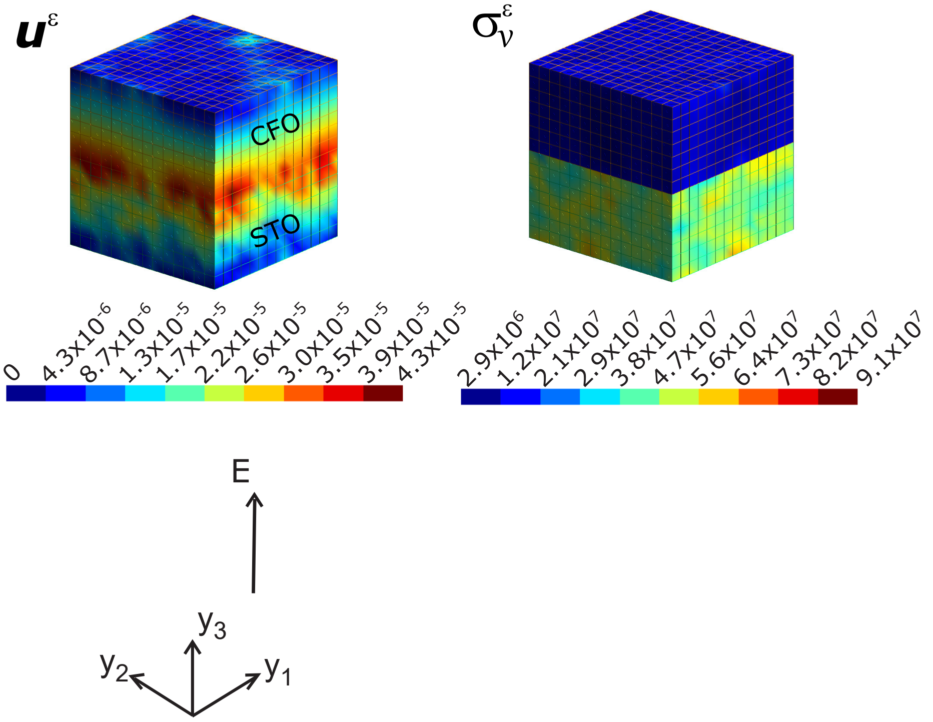

Figure S6. Map of the equivalent von Mises stress σ_v^ε (N/m^2), and displacement $\mathbf{u}^\varepsilon$ (m) (computed at the nodal points of the FEM) upon applying a global electric field $\mathbb{E}$ on the unit cell of magnetoelectric composite $SrTiO_3$–$CoFe_2O_4$.

of relatively few high peaks and low values. In case of printed films, skewness is a measure of variation of surface. Here, for STO skewness is measured to be -0.443. The negative value for skewness generally indicates that surface distribution has a longer lower valley at the measurement areas when compared to reference plane.

This study considers multiferroic materials that respond linearly to changes in the electric field, electric displacement, mechanical stress, strain as well as magnetic field. Let $\mathbf{\Omega}$ be a fixed domain in $\mathbf{x}$–space. We consider an auxiliary $\mathbf{y}$–space divided into parallelepiped periods $\mathbf{Y}$. For a linear anisotropic magnetoelectric material, generated through the periodic repetition of a *unit cell* representing the smallest sample of heterogeneity of the material domain $\mathbf{\Omega}$, the governing equations are given below;
force equilibrium equation,

$$\sigma_{ij,j} + b_i = \rho \ddot{u}_i \tag{S1}$$

strain-mechanical displacement relation,

$$\epsilon_{ij} = \frac{1}{2}(u_{i,j} + u_{j,i}), \tag{S2}$$

electrical (magnetic) field-electrical (magnetic) potential relations

$$\left. \begin{aligned} E_i &= -\varphi_{,i} \\ H_i &= -\psi_{,i} \end{aligned} \right\} \tag{S3}$$

and the quasistatic steady-state Maxwell's equations for electromagnetic phenomena,

$$\left. \begin{aligned} D_{i,i} &= 0 \\ B_{i,i} &= 0 \end{aligned} \right\} \tag{S4}$$

the mechanical, electrical and magnetic variables are related by constitutive relations. For small deformations, the linear constitutive laws of multiferroics in the absence of heat flux are given by

$$\sigma_{ij} = C^{EH}_{ijkl}\epsilon_{kl} - e_{kij}E_k - e^M_{kij}H_k \tag{S5}$$

$$D_i = e_{ijk}\epsilon_{jk} + \kappa^{\epsilon H}_{ij}E_j + \alpha_{ij}H_j \tag{S6}$$

$$B_i = e^M_{ijk}\epsilon_{jk} + \alpha_{ji}E_j + \mu^{\epsilon E}_{ij}H_j \tag{S7}$$

Here σ, ϵ, $\mathbf{u}$, $\mathbf{b}$, ρ, $\mathbf{D}$, $\mathbf{B}$ are stress, strain, displacement, body force, mass density, electric displacement vector, and magnetic flux density respectively. $\mathbf{C^{EH}}$, $\mathbf{e}$, $\mathbf{e^M}$, $\kappa^{\epsilon\mathbf{H}}$ and $\mu^{\epsilon\mathbf{E}}$ are stiffness, strain to (electric, magnetic) field coupling constants (or piezo-electric and -magnetic coefficients), permittivity (dielectric) and (magnetic) permeability respectively.

III. THEORY

Considering the standard homogenization procedure, the material functions $\mathbf{C^{EH}}$, $\mathbf{e}$, $\mathbf{e^M}$, $\kappa^{\epsilon\mathbf{H}}$ and $\mu^{\epsilon\mathbf{E}}$, are considered to be $\mathbf{Y}$-periodic functions in the unit cell domain defined as

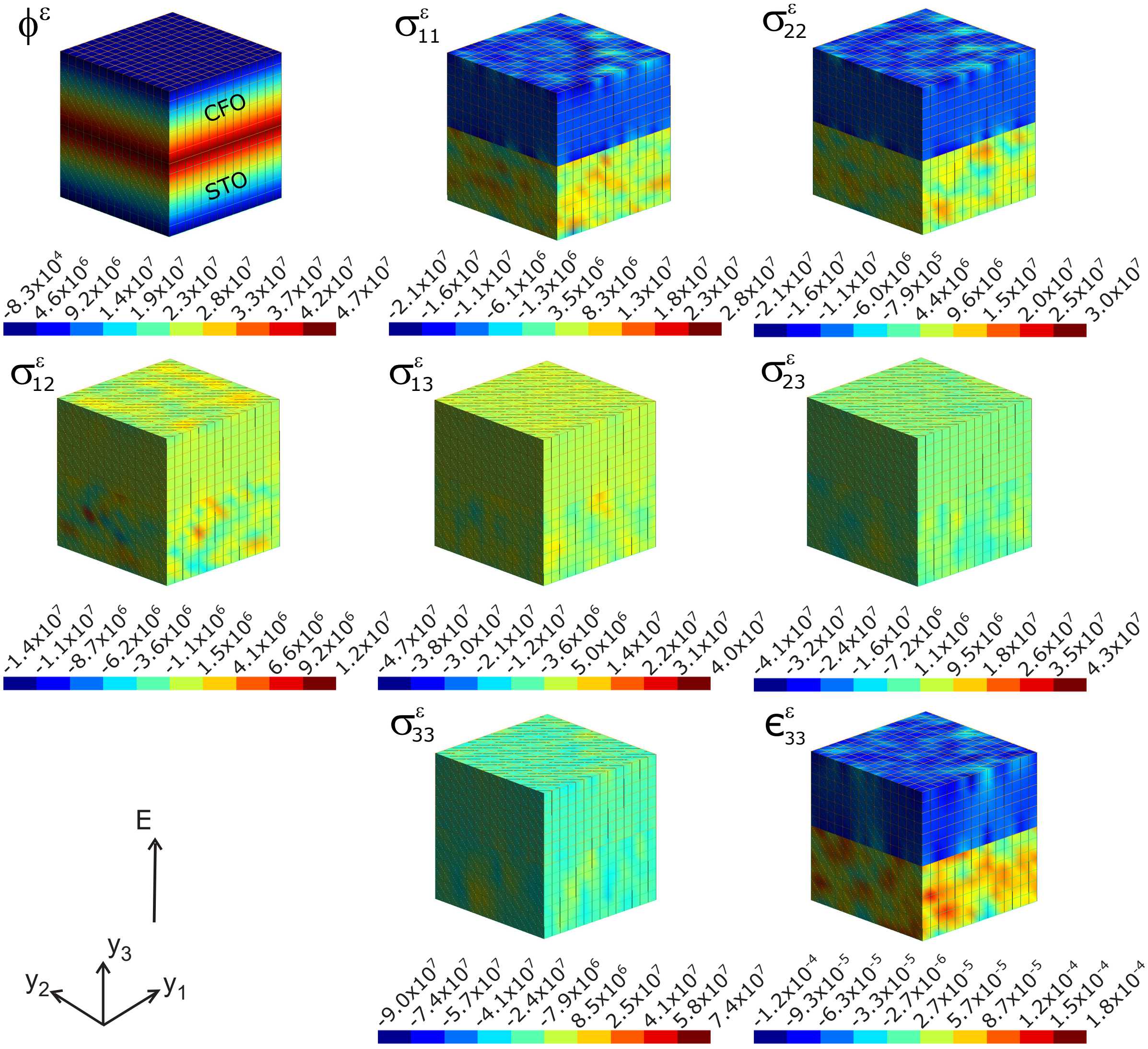

Figure S7. Map of local fields (computed at the nodal points of the FEM) of magnetoelectric composite $SrTiO_3$–$CoFe_2O_4$, *viz.*, electric potential φ^ε (V), the stress σ_{ij}^ε (N/m^2), the longitudinal strain $\epsilon_{33}^\varepsilon$, upon applying a global electric field $\mathbb{E}$ on the microstructure.

$\mathbf{Y} = [0, Y_1] \times [0, Y_2] \times [0, Y_3]^3$. If $\mathbf{y} = \mathbf{x}/\varepsilon$ where ε is the asymptotic scale factor representing the microstructure scale, the displacement $\mathbf{u}$, electric potential φ and magnetic potential ψ

are expanded asymptotically up to the first-order variation terms, giving

$$\left.\begin{aligned} \mathbf{u}^\varepsilon(\mathbf{x}) &= \mathbf{u}^0(\mathbf{x},\mathbf{y}) + \varepsilon \mathbf{u}^1(\mathbf{x},\mathbf{y}) \\ \varphi^\varepsilon(\mathbf{x}) &= \varphi^0(\mathbf{x},\mathbf{y}) + \varepsilon \varphi^1(\mathbf{x},\mathbf{y}) \\ \psi^\varepsilon(\mathbf{x}) &= \psi^0(\mathbf{x},\mathbf{y}) + \varepsilon \psi^1(\mathbf{x},\mathbf{y}) \end{aligned}\right\}, \mathbf{y} = \mathbf{x}/\varepsilon, \tag{S8}$$

where $\mathbf{u}^1(\mathbf{x},\mathbf{y}), \varphi^1(\mathbf{x},\mathbf{y})$ and $\psi^1(\mathbf{x},\mathbf{y})$ are functions to be determined which are $\mathbf{Y}$- periodic in $\mathbf{y}$. Here the two scales $\mathbf{x}$ and $\mathbf{y}$ are spatial variables where $\mathbf{x}$ is a macroscopic quantity and $\mathbf{y}$ is a microscopic one[4]. The functions involved in this expansion are assumed to be dependent on these two variables, where one (i.e., $\mathbf{x}$) describing the "global" or average response of the structure and the other (i.e., $\mathbf{y}$) describing the "local" or microstructural behaviour. here the two variables $\mathbf{x}$ and $\mathbf{x}/\varepsilon$ take into account the two scales of the homogenization; the $\mathbf{x}$ variable is the macroscopic variable, whereas the $\mathbf{x}/\varepsilon$ variable takes into account the *microscopic* geometry.

Applying calculus of variations, passing through the limit $\varepsilon \to 0$ and advance using asymptotic analysis one obtains the effective magneto-electro-elastic moduli, *viz.,* the homogenized elastic stiffnesses $\widetilde{C}^{EH}_{ijkl}$, piezoelectric coefficients $\widetilde{e}_{ijk}$, piezomagnetic coefficients $\widetilde{e}^{M}_{ijk}$, dielectric permittivities $\widetilde{\kappa}^{\epsilon H}_{ij}$, magnetic permeabilities $\widetilde{\mu}^{\epsilon E}_{ij}$ and the magnetoelectric coupling coefficients $\widetilde{\alpha}_{ij}$ of the magnetoelectric multiferroic. The detailed theoretical analysis is given elsewhere[3]. (In all the expressions of this paper, it may be noticed to discern the difference in notations of asymptotic scale factor ε and the mechanical strain ϵ. All the microscopic quantities will carry the notation of ε to identify their local character).

The displacement field $\mathbf{u}^\varepsilon(\mathbf{x})$, the electric potential field $\varphi^\varepsilon(\mathbf{x})$ and the magnetic potential field $\psi^\varepsilon(\mathbf{x})$ involving details of the microstructure are given by

$$\begin{aligned} \mathbf{u}^\varepsilon(\mathbf{x}) &= \mathbf{u}^0(\mathbf{x}) + \varepsilon\big[\chi^{mn}_k(\mathbf{x},\mathbf{y}) \\ &\quad \times \epsilon_{mn}(u^0(\mathbf{x})) + \Phi^m_k(\mathbf{x},\mathbf{y})\frac{\partial \varphi^0(\mathbf{x})}{\partial x_m} \\ &\quad + \Gamma^m_k(\mathbf{x},\mathbf{y})\frac{\partial \psi^0(\mathbf{x})}{\partial x_m}\big] \end{aligned} \tag{S9}$$

TABLE S1. Values of the homogenized piezoelectric stress coefficients $\widetilde{e}_{i\mu}$ (in C/m^2), piezomagnetic coefficients $\widetilde{e}_{i\mu}^{M}$ (in N/Am) and dielectric permittivity $\widetilde{\kappa}_{ij}^{\epsilon H}$ (in κ_0), ME coupling $\widetilde{\alpha}_{11}$ (in 10^{-8} Ns/VC), $\widetilde{\alpha}_{33}$ (in 10^{-10} Ns/VC), and ME voltage coefficient (absolute value) $\widetilde{\alpha}_{E11}$ (in mV/cmOe) and α_E (in mV/cmOe) of single crystal ME composite STO–CFO. (κ_0 is the permittivity of free space)

$\widetilde{e}_{31}$	$\widetilde{e}_{33}$	$\widetilde{e}_{24}$	$\widetilde{e}_{31}^{M}$	$\widetilde{e}_{33}^{M}$	$\widetilde{e}_{24}^{M}$	$\widetilde{\kappa}_{11}^{\epsilon H}$	$\widetilde{\kappa}_{33}^{\epsilon H}$	$\widetilde{\alpha}_{11}$	$\widetilde{\alpha}_{33}$	$\widetilde{\alpha}_{E11}$	α_E
0.04	0.26	1.32	30.4	44.5	400.2	121.6	20.4	-0.8	-0.41	5.94	0.18

.

$$\begin{aligned}\varphi^{\varepsilon}(\mathbf{x}) &= \varphi^{0}(\mathbf{x}) + \varepsilon\big[\eta^{mn}(\mathbf{x},\mathbf{y}) \\ &\quad \times\epsilon_{mn}(u^{0}(\mathbf{x})) + R^{m}(\mathbf{x},\mathbf{y})\frac{\partial\varphi^{0}(\mathbf{x})}{\partial x_m} \\ &\quad +\Psi^{m}(\mathbf{x},\mathbf{y})\frac{\partial\psi^{0}(\mathbf{x})}{\partial x_m}\big] \end{aligned} \tag{S10}$$

$$\begin{aligned}\psi^{\varepsilon}(\mathbf{x}) &= \psi^{0}(\mathbf{x}) + \varepsilon\big[\lambda^{mn}(\mathbf{x},\mathbf{y}) \\ &\quad \times\epsilon_{mn}(u^{0}(\mathbf{x})) + \Theta^{m}(\mathbf{x},\mathbf{y})\frac{\partial\varphi^{0}(\mathbf{x})}{\partial x_m} \\ &\quad +Q^{m}(\mathbf{x},\mathbf{y})\frac{\partial\psi^{0}(\mathbf{x})}{\partial x_m}\big] \end{aligned} \tag{S11}$$

The local strain $\epsilon_{ij}^{\varepsilon}(\mathbf{x})$, electric field $E_j^{\varepsilon}(\mathbf{x})$ and the magnetic field $H_j^{\varepsilon}(\mathbf{x})$ too can be obtained in a similar way once the homogenized macroscopic problem is solved. Here χ, R and Q are microscopic characteristic material, electric and magnetic displacements respectively. $\mathbf{\Phi}$, $\mathbf{\Gamma}$, η,Ψ,λ and Θ are characteristic coupled functions. It is postulated that $\mathbf{u}^0$ is constant with respect to $\mathbf{y}$, and depends only on $\mathbf{x}$ and hence can be equally valid for other fields φ^0 and ψ^{0}[4]. The asymptotic expansion in Eq. S11 means that the fields $\mathbf{u}^{\varepsilon}$, φ^{ε} and ψ^{ε} are the smooth functions plus the trailing little perturbing terms.

IV. NUMERICAL IMPLEMENTATION

The methodology used to obtain the average and local fields is based on the software POSTMAT(*material postprocessing*) developed by Guedes and Kikuchi[5]. It is to be noticed that the homogenized coefficients only depend on the local (microscopic) structure of the medium, and is obtained by the numerical solution of the boundary value problem, where

TABLE S2. Magnetoelectric properties of lead free ferroelectric-ferromagnetic ceramic composites with 2–2 connectivity.

**Sl No**	**Composite**	**Connectivity scheme**	**α_{ME}**	**Method**	**Reference**
1	$BaTiO_3/CoFe_2O_4$	Laminate ceramics	135 (mV $cm^{-1}.Oe^{-1}$)	Cold isostatic pressing	J. All. Comp., 644, 390-397 (2015)
2	$0.5(Ba_{0.7}Ca_{0.3}TiO_3)$-$0.5(BaZr_{0.2}Ti_{0.8}O_3)$ $/CoFe_2O_4$	Bilayer Thin film	105 (mV $cm^{-1}.Oe^{-1}$)	Solution deposition	J. Appl. Phys., 120, 74108 (2016)
3	$Ba_{0.85}Ca_{0.15}Zr_{0.1}Ti_{0.9}O_3$ $/CoFe_2O_4$	Bilayer Laminates	320 (mV $cm^{-1}.Oe^{-}1$)	Solution deposition	J. Inorg. Mater., 28, pp. 317-320 (2013)
4	$(Ba_{0.85}Ca_{0.15})(Zr_{0.1}Ti_{0.9})O_3$ $/CoFe_2O_4$	Bilayer Laminate	615 (mV $cm^{-1}.Oe^{-1}$)	Ceramics with epoxy bonding	Ceram. Inter., 44(4), 4298-4306 (2018)
5	$BaTiO_3/CoFe_2O_4$	Bilayer laminate	8.1 (µV $cm^{-1}.Oe^{-1}$)	Tape Casting	J. Mat. Sci., 48, 178–185, (2013)
6	$BaTiO_3/CoFe_2O_4$	Bilayer laminate	36 (µV $cm^{-1}.Oe^{-1}$)	Tape Casting	J. Mater. Sci.-Mater. Electron., 23, 2098–2103,(2012)
7	NiFe2O4/BaTiO3	Bilayer laminate	18 (mV $cm^{-1}.Oe^{-1}$)	Tape Casting	Appl. Phy. Express 4 073001(2011)
**8**	**Pr:$SrTiO_3/CoFe_2O_4$**	**Bilayer laminate**	**779 (mV cm^{-1}. Oe^{-1})**	**Screen Printing**	**This work**

the boundary conditions being of the periodic type. The homogenization method gives relevant information on the local and global behaviours in contrast to majority of problems in mechanics where the micro- and macro-processes are of very different nature[4]. Microscopic cell problems are solved prior to the macroscopic homogenized ones, and the finite element approximations related to the microscopic problem are defined to evaluate the homogenized coefficients subjected to periodic boundary conditions. Details of numerical implementation of homogenization magnetoelectric composite are briefed below. Once the homogenized solutions for for $\mathbf{u}^0$, φ^0 and ψ^0 are known then one can compute, the local displacement, potentials, fields, strains, stresses, and equivalent von-Mises stresses for each element and at each integration point. (The equivalent von-Mises stress at the nodes is an average of the values at the Gauss points of the surrounding elements with same material properties.)

Magnetoelectric multiferroic material, in general, can be considered as an aggregate of single crystalline crystallites/grains and hence the system altogether would be polycrystalline nature. The unit cell or the representative volume element (RVE) conceived in this work is a volume containing a sufficiently large number of crystallites or grains that its properties can be considered as equivalent to that of the macroscopic sample. The electric polarization $\mathbf{P}$ as well as the magnetization $\mathbf{M}$ interspersed inside the crystallites could be mapped using using some coordinate system. In a sense the underlying crystal orientation can encompass the orientations of $\mathbf{P}$ or $\mathbf{M}$. Thus we introduce the Euler angles (φ, θ, ψ) to quantify the crystal orientations of a multiferroic polycrystal, as the crystallites in an as-grown sample are randomly oriented in the lattice space and hence require three angles to describe its orientation with reference to a fixed coordinate system. Here we use the so called *x-convention*, where the first and third rotation is through the y-axis (here it is y_2'–axis)and the second rotation is through the intermediate x-axis (here it is y_1'–axis). Thus all the physical quantities $\lambda'_{ijklmn...}(\mathbf{y}')$ expressed in a crystallographic coordinate system $\mathbf{y}'$ would be coordinate-transformed to the local coordinate system $\mathbf{y}$ according to the following scheme

$$\lambda_{ijkl...}(\mathbf{y}) = e_{im}e_{jn}e_{kp}e_{lq}\ldots\widetilde{\lambda'}_{mnpq...}(\mathbf{y}') \tag{S12}$$

before it is introduced for homogenization. (i.e., the FE and FM materials' electromechanical

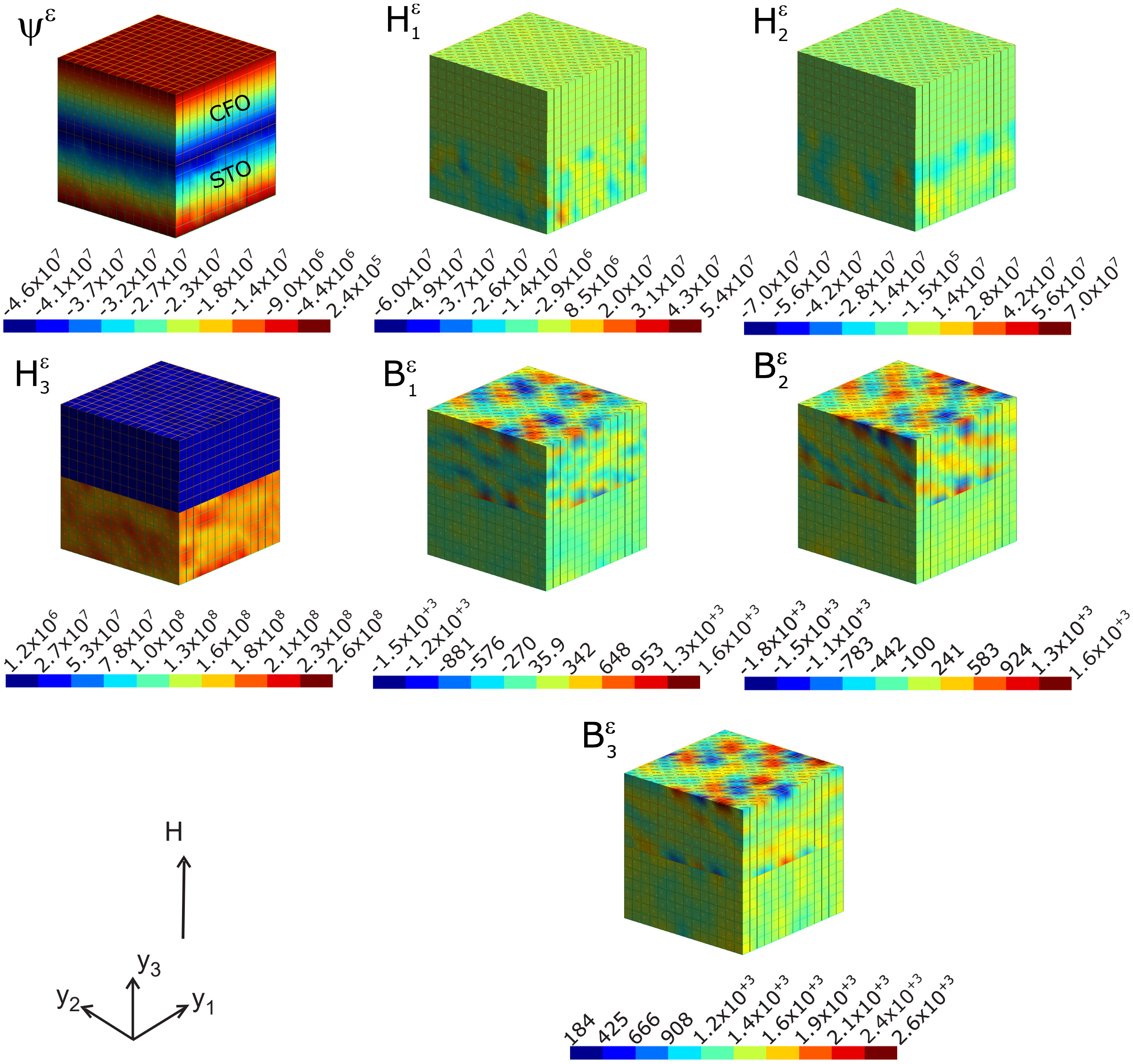

Figure S8. Map of local magnetic scalar potential ψ^ε (A), magnetic field H^ε_j (A/m), and magnetic flux B^ε_j (Wb/m^2) computed at the nodal points of the FEM, upon applying a biasing magnetic field $\mathbb{H}$ on a magnetoelectric composite of $SrTiO_3$–$CoFe_2O_4$.

property data entered into the homogenization program are obtained with respect to the crystallographic coordinates.) Here $e_{\mu\nu}$ are the Euler transformation matrices[6].

In the FEM simulation of the homogenization, a multiferroic crystallite is represented by a finite element in the unit cell. Thus we have a polycrystalline unite cell having as much number of crystallites as the number of finite elements by which it is discretized. As-grown FE (or for that matter FM) polycrystal, often ends up in a near complete compensation

of polarization (or magnetization) and the material consequently exhibit very small, if any, electric (or magnetic) effect until they are poled by the application of an electric (magnetic) field. The orientation distribution of the crystallites (grains) in such a polycrystalline material would be uniform with a standard deviation $\sigma \to \infty$ before poling (application of electric/magnetic field) and that after poling would best be represented by a distribution function with $\sigma \to 0$. Thus, any pragmatic configuration of orientation distribution of grains in multiferroic material would fit in a Gaussian distribution defined by the probability distribution function

$$f(\alpha \mid \mu, \sigma) = \frac{1}{(\sigma\sqrt{2\pi})} \exp -\Big[\frac{(\alpha-\mu)^2}{2\sigma^2}\Big] \tag{S13}$$

where μ and σ are the the mean and the standard deviation of the angles α (which stands for the Euler angles $(\phi, \theta, \vartheta)$). The convergence of magnetoelectric properties with unit cell size allows us to determine the simulation-space independent, equivalent magnetoelectric properties of the composite. Convergence analyses, on magnetoelectric composites reveal that accuracy one derives from descretizing the unit cell (in other words sampling of the unit cells with more number of grains or less number) is minimal above 1000 elements (grains)[7]. The detailed plots were given in the Supplementary materials of the same Ref.[7]. Consequently, we kept unit cells' sizes greater than 1000 finite elements in this study.

The microscopic system of equations resulting from homogenization has been solved using a finite element (FEM) formulation[3]. A three-dimensional (3D) multiferroic finite element is conceived with five degrees of freedom (DOF)-three DOFs for spatial displacements and one each for electric and magnetic potentials. Eight-noded isoparametric elements with $2 \times 2 \times 2$ Gauss-point integration are used obtain solutions. Altogether there were nine microscopic equations that should be solved for as much number of unknowns namely the characteristic functions $\chi_i^{mn}, \eta^{mn}, \lambda^{mn}, R^m, \Phi_i^m, \Theta^m, Q^m, \Psi^m$ and Γ_k^m, where the indices $\mathrm{m}, \mathrm{n} = 1, 2, 3$[3]. The problem is reduced to standard variational FEM, after the usual approximations of finite element formulation and can be expressed concisely as

$$\boldsymbol{K}\boldsymbol{u} = \boldsymbol{f} \tag{S14}$$

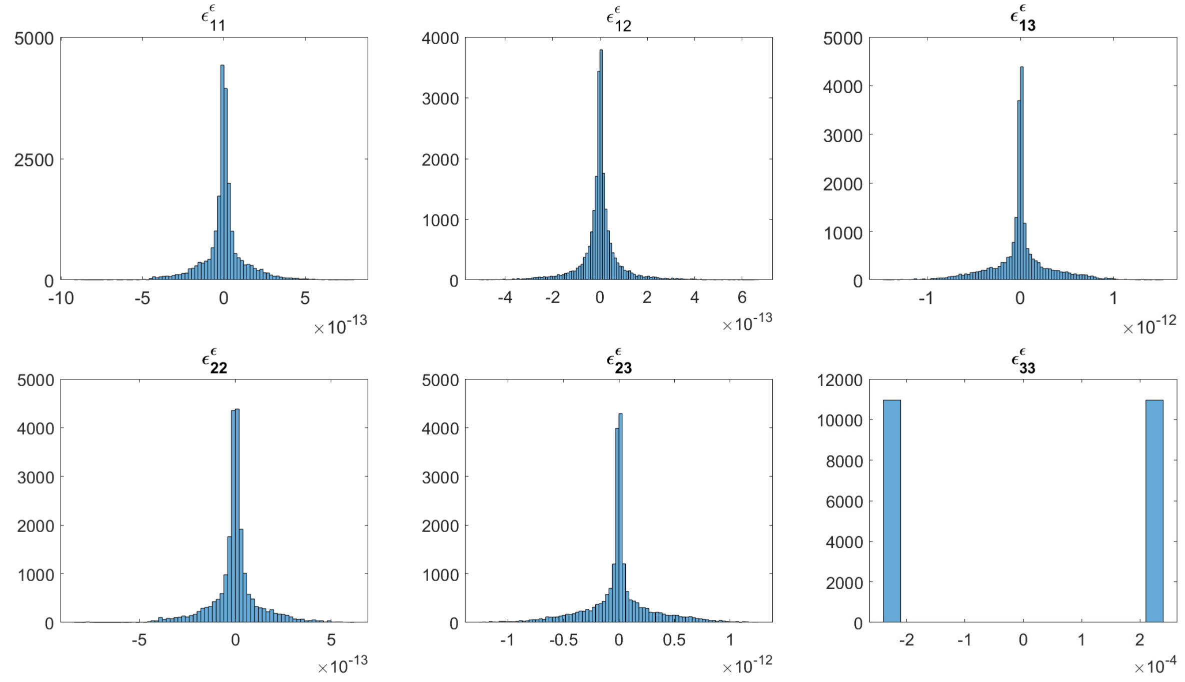

Figure S9. Histogram of the local strain ($\epsilon^{\varepsilon}_{ij}$) values consequent to the application of external magnetic field (averaged over finite elements) on single crystal STO–CFO composite.

where $\boldsymbol{K}$ is the global stiffness matrix, $\boldsymbol{u}$ is the vector of unknown functions and $\boldsymbol{f}$ is the load vector.

The work flow of the computational procedure is summarised in the flowchart give in Fig. S5. for further details regarding the computation and the terms mentioned in the flowchart, please refer to the Refs.[3,8]

V. RESULTS

The homogenization was run initially to see the magnetoelectric properties of the single crustal STO–CFO composite. The property values obtained have been given in Table S S1.

So far as screen print generated lead free ferroelectric-ferromagnetic ceramic composites with 2-2 connectivity are concerned, to the best of our knowledge, there is no reports available dealing with their magnetoelectric coupling. However, there are reports on the bi-layer laminates generated through other techniques like tape casting, solution deposition, laminate

composites generated through cold isostatic pressing etc., which is tabulated in the table **??** below. Evidently, the present report outperforms the reported literature on bi-layer FE-FM all-ceramic laminates.

The simulation results of distribution of local (*microscopic*) fields *viz.*, the equivalent, von Mises stress σ_v^ε and displacement $\mathbf{u}^\varepsilon$ consequent to the application of an external fields along the y_3–axis of the composite microstructure are displayed in Figs. S6, S7 and S8.

REFERENCES

[1] V. Senthil, J. Gajendiran, S. G. Raj, T. Shanmugavel, G. Ramesh Kumar, C. Parthasaradhi Reddy, Study of structural and magnetic properties of cobalt ferrite (cofe2o4) nanostructures, Chem Phys Lett 695 (2018) 19–23. doi:doi:10.1016/j.cplett.2018.01.057.

[2] J. F. Scott, Ferroelectrics go bananas, J Phys- Condens Mat 20 (2007) 021001. doi:doi:10.1088/0953-8984/20/02/021001.

[3] K. P. Jayachandran, J. M. Guedes, H. C. Rodrigues, A generic homogenization model for magnetoelectric multiferroics, J Intel Mat Syst Str 25 (2014) 1243–1255. doi:doi:10.1177/1045389X13502877.

[4] E. Sanchez-Palencia, Non-homogeneous media and vibration theory, Lecture notes in physics 127, Springer-Verlag, Berlin, 1980.

[5] J. M. Guedes, N. Kikuchi, Preprocessing and postprocessing for materials based on the homogenization method with adaptive finite element methods, Comput. Methods Appl. Mech. Eng. 83 (1990) 143–198.

[6] H. Goldstein, Classical Mechanics, Addison-Wesley, Reading, MA, 1978.

[7] K. P. Jayachandran, J. M. Guedes, H. C. Rodrigues, Solutions for maximum coupling in multiferroic magnetoelectric composites by material design, Sci. Rep. 8 (2018) 4866. doi:doi:10.1038/s41598-018-22964-9.

[8] K. P. Jayachandran, J. M. Guedes, H. C. Rodrigues, Homogenization method for microscopic characterization of the composite magnetoelectric multiferroics, Sci Rep 10 (2020) 1276. doi:doi:10.1038/s41598-020-57977-w.
